# Additively manufactured Ti–Ta–Cu alloys for the next-generation load-bearing implants

**DOI:** 10.1088/2631-7990/ad07e7

**Published:** 2023-11-17

**Authors:** Amit Bandyopadhyay, Indranath Mitra, Sushant Ciliveri, Jose D Avila, William Dernell, Stuart B Goodman, Susmita Bose

**Affiliations:** 1 W. M. Keck Biomedical Materials Research Laboratory, School of Mechanical and Materials Engineering, Washington State University, Pullman, WA 99164, United States of America; 2 Veterinary Clinical Sciences, College of Veterinary Medicine, Washington State University, Pullman, WA 99164, United States of America; 3 Department of Orthopedic Surgery, Stanford University Medical Center, Redwood City, CA 94063, United States of America

**Keywords:** Ti6Al4V, load-bearing implants, additive manufacturing, 3D printing, antibacterial performance

## Abstract

Bacterial colonization of orthopedic implants is one of the leading causes of failure and clinical complexities for load-bearing metallic implants. Topical or systemic administration of antibiotics may not offer the most efficient defense against colonization, especially in the case of secondary infection, leading to surgical removal of implants and in some cases even limbs. In this study, laser powder bed fusion was implemented to fabricate Ti3Al2V alloy by a 1:1 weight mixture of CpTi and Ti6Al4V powders. Ti-Tantalum (Ta)–Copper (Cu) alloys were further analyzed by the addition of Ta and Cu into the Ti3Al2V custom alloy. The biological, mechanical, and tribo-biocorrosion properties of Ti3Al2V alloy were evaluated. A 10 wt.% Ta (10Ta) and 3 wt.% Cu (3Cu) were added to the Ti3Al2V alloy to enhance biocompatibility and impart inherent bacterial resistance. Additively manufactured implants were investigated for resistance against *Pseudomonas aeruginosa* and *Staphylococcus aureus* strains of bacteria for up to 48 h. A 3 wt.% Cu addition to Ti3Al2V displayed improved antibacterial efficacy, i.e. 78%–86% with respect to CpTi. Mechanical properties for Ti3Al2V–10Ta–3Cu alloy were evaluated, demonstrating excellent fatigue resistance, exceptional shear strength, and improved tribological and tribo-biocorrosion characteristics when compared to Ti6Al4V. *In vivo* studies using a rat distal femur model revealed improved early-stage osseointegration for alloys with 10 wt.% Ta addition compared to CpTi and Ti6Al4V. The 3 wt.% Cu-added compositions displayed biocompatibility and no adverse inflammatory response *in vivo*. Our results establish the Ti3Al2V–10Ta–3Cu alloy’s synergistic effect on improving both *in vivo* biocompatibility and microbial resistance for the next generation of load-bearing metallic implants.

## Highlights

Ti3Al2V demonstrated comparable mechanical performance to Ti6Al4V.
Adding 3 wt.% Cu in Ti3Al2V reduced planktonic bacteria colonies by 78%–86% compared to commercially pure Ti.
Ti3Al2V–10Ta displayed the best *in vivo* biocompatibility with 3.5-fold higher bone formation than Ti6Al4V.
Ti3Al2V–10Ta–3Cu multifaceted alloy has the potential to replace Ti6Al4V in orthopedic and dental applications with superior early-stage osseointegration and inherent antibacterial performance.

## 1. Introduction

Early-stage osseointegration is one of the most desirable qualities of metallic implants as it promotes faster healing and long-term implant stability. Early-stage osseointegration primarily depends on an implant’s biocompatibility. Compromised biocompatibility due to bacterial growth on the implant surface has been shown to result in adverse events such as septic loosening and prosthetic joint infection (PJI), which ultimately require revision surgeries to mitigate such clinical challenges [1, 2]. Growth in the incidence of revision surgeries due to PJI is projected to increase to 176% and 170% for total hip arthropathy (THA) and total knee arthropathy (TKA) by 2030, supporting prior literature suggesting a losing battle against this postoperative outcome. A recent World Health Organization report stated that around 700,000 deaths occur yearly due to antimicrobial resistance. If clinical remedies are not found, as many as 10 million deaths per year are predicted by 2050 due to infection, higher than 8.2 million deaths per year due to cancer, and will become a significant economic burden worldwide [3]. In addition, the mortality rate for PJI is 87.3%, which is greater than that for colorectal and lung cancer and comparable to those for breast cancer (89%) [4, 5]—making PJI a compelling and critical clinical challenge that needs immediate attention. Available therapy is based on a two-pronged approach of (1) extensive local debridement and implant replacement via revision surgery and (2) antibiotic treatments at the local surgery site or through systemic administration. However, those approaches are not always sustainable, leading to recurring infections even after revision surgery [6–9]. Our research addresses this concern by fortifying titanium (Ti) implants with 3 wt.% copper (Cu) via multi-material additive manufacturing to inherently protect the implant against bacterial infection while enhancing biocompatibility by adding tantalum (Ta) to facilitate early-stage osseointegration.

The mutual exclusivity of high infection rate (postoperative spine infection 0%–18% and knee/hip arthroplasty 1%–2%) [10], revision surgeries (8%–15% for arthroplasty), and out-of-pocket costs associated with such procedures (up to $93,000 in 2009) further complicate the problem [11]. The most common infections originate from *Staphylococcus aureus* (*S. aureus* ∼66%) or *Pseudomonas aeruginosa* (*P. aeruginosa* ∼15%) [12], causing a recurrent infection rate for *S. aureus* following revision surgeries as high as 75%, while only 56% are cured at 1 year post-op [13]. In addition, infection of diverse types of implants such as hip, knee, and spine require highly heterogeneous modes of treatment, which can make healthcare complicated and expensive. The heterogeneity is heavily biased towards material evaluation such as *in vivo* failure analyses, biological response, and implant success with respect to material properties. Therefore, implants should be self-sufficient in preventing PJI and mitigating the complexities of revision surgeries. Multifunctional materials such as Ti–Cu alloys can ensure implant success, reduce healthcare costs, and increase value-of-product. Currently, most studies have evaluated Ti implants alloyed with ⩾5 wt.% Cu for bacterial resistance fabricated via powder metallurgy [14]. However, the general perspective on such high amounts of Cu addition in implant materials is linked to scientific concerns. Moreover, these studies do not account for any cytotoxicity from higher amounts of Cu or evaluate pathways for comprehensively improving the early-stage osseointegration ability of the implants.

Our research aims to evaluate persistent bacterial resistance and early-stage osseointegration of additively manufactured Ti-3 wt.% Cu alloy implants. We hypothesize that alloying as low as 3 wt.% Cu would make the implants bacteria-resistant long-term, leading to enhanced early-stage osseointegration. Aiming to develop a first-generation alloy with an improved biological response than Ti6Al4V and comparable mechanical properties to Ti6Al4V, we ask a simple question: *do we need 6% Aluminum and 4% Vanadium in Ti for load-bearing implants?* Instead of developing ternary Ti-alloys with multiple stoichiometric fractions of alloying elements, we provide a simple solution by reducing the amount of aluminum (Al) and vanadium (V) in Ti6Al4V to fabricate Ti3Al2V for enhanced biocompatibility. Additionally, we incorporated 10 wt.% Ta to the Ti3Al2V-3 wt.% Cu alloys to modify the primary material and mitigate compromised biocompatibility (if any) due to Cu addition. Therefore, our material can resist bacterial invasion and enhance osseointegration for faster healing and bone regeneration.

**Figure 1.**
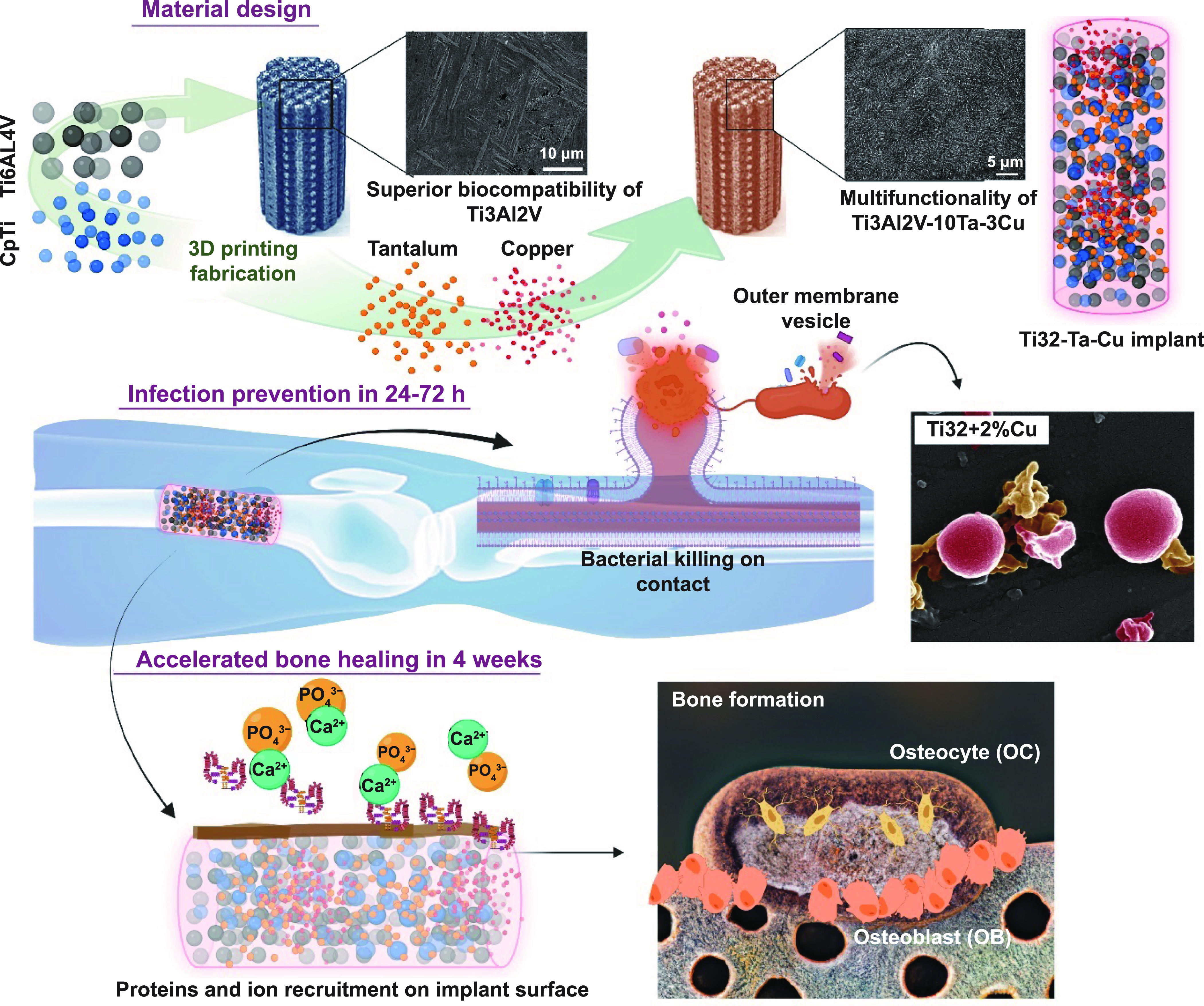
Schematic of synergistic antibacterial efficacy and enhanced biocompatibility of Ti3Al2V–Cu–Ta alloys toward early-stage osseointegration. These implants are fabricated via multi-material additive manufacturing to impart continued bacterial resistance while enabling early bone formation through enhanced biocompatibility.

In this light, we have fabricated Ti3Al2V by physically pre-mixing CpTi and Ti6Al4V powder in equal weight proportions using metal additive manufacturing to evaluate the mechanical and biological response of processed dense and porous parts. Following this, multi-material additive manufacturing was employed to alloy Ti3Al2V with 2 and 3 wt.% Cu for antibacterial efficacy evaluation. Finally, enhancement in biological response and early-stage osseointegration was assessed by incorporating 10 wt.% Ta in the Ti3Al2V–Cu alloys based on our previous report that showed 10 wt.% Ta addition in CpTi can significantly improve the biological response of Ti alloys [15] (figure 1). By exploiting the intrinsic material flexibility associated with additive manufacturing and thus enabling material design to incorporate functionalities for bacterial resistance, our research takes us one step further toward more efficient individualized therapy.

## 2. Materials and methods

### 2.1. Design and fabrication: selective laser melting (SLM)

All structures were fabricated on a SLM-based powder bed fusion (PBF) system (3D Systems ProX^®^ DMP 200, Rock Hill, SC) with a 300 W fiber laser and wavelength *λ* = 1070 nm. The system has a powder supply chamber and a melting stage. Spherical metal powders were used. CpTi powders were procured from GKN Hoeganaes (Cinnaminson, NJ, USA) and Ti6Al4V from AP&C (GE Additive, Cincinnati, Ohio, USA). Metal powders were sieved to obtain a particle size of <63 *μ*m. These metal powders were added to the supply side. A CpTi build plate of ∼2.5 cm thickness was used and placed on the melting stage. The build chamber was enclosed and purged with argon gas with O_2_ < 500 ppm. CpTi and Ti6Al4V compositions were printed on the laser PBF (LPBF) system. A third composition was prepared and printed by mixing CpTi and Ti6Al4V powders in a 1:1 weight ratio, called Ti3Al2V, since the amounts of Al and V were reduced by 50% each to 3 and 2 wt.%, respectively. Individual dense and porous (20% and 40% volume fraction porosity) cylindrical structures were designed for compression and shear strength measurements at the porous-dense interface. Alloy compositions Ti3Al2V–2Cu and -3Cu were prepared by mixing 2 and 3 wt.%s of Cu powders (GKN Hoeganaes, Cinnaminson, NJ, USA) in premixed Ti3Al2V, respectively. Ti3Al2V–10Ta was prepared by mixing 10 wt.% Ta (Tekna, Sherbrooke, Québec, Canada) in Ti3Al2V. Finally, Ti3Al2V–10Ta–3Cu was prepared by mixing 10 wt.% Ta (10Ta) and 3 wt.% of Cu (3Cu), in Ti3Al2V. Alloy compositions Ti3Al2V–2Cu, Ti3Al2V–3Cu, Ti3Al2V–10Ta, and Ti3Al2V–10Ta–3Cu were built using LPBF for *in vitro* bacterial resistance studies (dense) and *in vivo* biological response studies (40% volume fraction porosity).

Additive manufacturing of Ti6Al4V and CpTi is widely used. The optimized laser power and scan speed parameters are 180 W and 1600 mm·s^−1^, respectively, for successful fabrication using our PBF process, which was used for compositions CpTi, Ti6Al4V, Ti3Al2V, Ti3Al2V–3Cu, and Ti3Al2V–10Ta–3Cu. With the addition of alloying elements, the part quality and print resolution varied depending on intrinsic material properties such as heat diffusivity and laser absorption. Ta and Cu demonstrate contrasting laser-material interactions. Ta shows excellent laser absorption but has a very high melting point of 3017 °C, indicating higher energy input for successful PBF. However, Cu is a poor laser absorber, reflecting 98% of the laser wavelengths in 1000–1100 nm range [16]. Cu also demonstrates more than two times the viscosity and 100 times heat diffusivity in the molten state compared to Ti, dictating the higher energy required for the laser-PBF operation [17, 18]. An in-depth study on the PBF printing optimization for these alloys resulted in a laser power of 196 W and a scanning speed of 1440 mm·s^−1^ to be the optimal printing parameters used to fabricate these compositions [19].

A layer thickness of 30 *μ*m was used for each layer. The hexagonal laser scan strategy was used for dense and strip laser scan strategy for printing porous structures. All compositions were printed with the same print parameters (denoted as ‘as-printed’ structures hereafter). Post printing, structures were cut from the build plate and ground on 120 grit SiC papers to make the opposite surfaces parallel, followed by multiple sonication treatments in deionized (DI) water and ethanol, then compressed air spraying to remove all loose powders inside the pores. Bulk volume porosities were calculated by taking the measured volume of the structures to the theoretical volume of their respective dense composition. Rectangular cross-section samples were manufactured for tribological-biocorrosion studies; these samples were fabricated on a directed energy deposition (DED)-based AM system (FormAlloy, CA) and described briefly in the supplemental file.

### 2.2. Phase analysis, microhardness, and microstructure

Phase detection was done using x-ray diffraction (XRD) analysis. Vickers microhardness tests were performed on the surface in the *x–y* plane of the build direction. Microstructure was observed by cutting longitudinally along the build direction. The sections were mounted in a phenolic resin followed by grinding on 80*–*1200 grit size SiC grinding papers and polishing using 0.05*–*1 *μ*m of suspended alumina in DI water. Vickers microhardness test was conducted on a Phase II Plus Micro Vickers Hardness tester (Upper Saddle River, NJ, USA) using a load of 200 g and a dwell time of 15 s. A total of *n* = 15 microhardness measurements were conducted for each composition. Surfaces were etched in Kroll’s reagent for 45 s, and microstructures were observed under a scanning electron microscope (SEM, Apreo, Thermo Scientific, MA, USA).

### 2.3. Mechanical strength evaluation: compression, shear, and fatigue

As per ASTM E9-19, dense and porous cylindrical structures for all compositions with 7 mm diameter and ∼15 mm height were subjected to compression testing on an Instron servo-hydraulic machine (600DXS, Grove City, Pennsylvania). A minimum of 3 replicates were tested for each composition and porosity. A crosshead displacement rate of 1.3 mm·min^−1^ was used across the compositions. The corresponding load*–*displacement data was recorded. Elastic modulus was calculated from the linear slope region from the stress*–*strain curve. Compressive yield strength was evaluated using the 0.2% strain offset method. Shear strength was evaluated at the porous-dense interface for Ti6Al4V and Ti3Al2V using a single-shear test device developed in our lab per the procedure described in [20, 21]. Structures with 3.1 mm diameter and ∼12 mm height were used. The nature of the shear test carried out is tensile rather than torsional. The porous-dense interface was placed at the interface of the shear plates and pulled in tension in opposite directions with a crosshead displacement rate of 0.3 mm·min^−1^ on an Instron servo-hydraulic testing machine (600DXS, Grove City, Pennsylvania). An external 1360 kg load cell and an extensometer were used to precisely measure the load and displacement of the shear plates, respectively. Structures were sheared till fracture, and the corresponding load and displacement data were recorded. At least three replicates were tested for each composition and porosity, respectively. The shear modulus was evaluated as the slope of the linear region in the shear stress*–*strain curve. Maximum shear strength was evaluated as the highest shear strength endured by the structure before failure. Fractured surfaces at the porous-dense interface were observed under a SEM (Apreo, Thermo Scientific, MA, USA). No etching was done on fracture surfaces for microstructural imaging. The fatigue tests were performed on an ADMET eXpert 9300—Rotating Beam Fatigue system (Norwood, MA). The Rotating Beam tester applies a force via a bending moment to induce surface stress on a sample. Each surface experiences tensile and compressive stresses as the sample rotates until failure. The digital controller displays the number of cycles for the sample to fail.

### 2.4. Wear and open circuit potential (OCP) tests

*In vitro* tribological-biocorrosive testing was carried out using a ball-on-flat set-up on ground-polished DED printed CpTi, Ti6Al4V, Ti3Al2V, Ti3Al2V–3Cu, Ti3Al2V–10Ta and Ti3Al2V–10Ta–3Cu samples following ASTM G133-05 [22]. The tests were done using a Biotribometer (Ducom, India) in Dulbecco’s Modified Eagle’s medium (DMEM) (Sigma-Aldrich) with a 5 N applied normal load, 3 mm diameter zirconia (ZrO_2_) wear ball, translation speed of 72 m·h^−1^, and a 10 mm amplitude for a total sliding distance of 1000 m. Compound wear (CW) and coefficient of friction (COF) were recorded; compound wear for the tested samples was attained using the built-in linear variable differential transformer (LVDT) to measure the *z*-axis displacement of the tribological loading arm throughout testing. The measurement considers both wear on the counter wear ball and the tested sample, hence the attained compound wear. A 2-electrode corrosion-cell configuration acquired the OCP with a modular line Metrohm Autolab potentiostat/galvanostat (Riverview, FL, USA). The fabricated structures were used as the working electrode (WE), and the reference electrode (RE) was a saturated Ag/AgCl/KCl. The structures were immersed in DMEM and allowed to stabilize for a minimum of 2–3 h before starting the tribological wear test. The tracks on the samples and scars on the ZrO_2_ wear balls were imaged under a SEM (Quanta 200F, Thermo Fisher, Waltham, MA) and optical microscope, respectively.

### 2.5. *In vivo* study

Both dense and porous (40% volume fraction porosity) CpTi, Ti6Al4V, Ti3Al2V, Ti3Al2V–3Cu, and Ti3Al2V–10Ta–3Cu compositions were used for the *in vivo* biological response study. *In vivo* studies were designed with a 2-phase parallel evaluation. First, CpTi and Ti6Al4V were considered controls to evaluate the biological response of Ti3Al2V compositions. This phase was designed to assess whether Ti3Al2V had similar or better osseointegration than the already established CpTi without compromising the mechanical tissue-material fixation that Ti6Al4V offers. The second phase was a post-Ti3Al2V assessment to evaluate toxicity, enhancement in biological response, and osseointegration of Ti3Al2V–3Cu and Ti3Al2V–10Ta–3Cu over Ti3Al2V.

#### 2.5.1. Surgery and implantation procedure.

Male Sprague–Dawley rats with average weights between 300 and 350 g were used for the *in vivo* study. The rats were acclimatized in separate cages in a temperature and humidity-controlled room for at least a week before the surgeries. The animals were administered buprenorphine (0.03 mg·kg^−1^) 30 min before anesthesia as a pain-reducing medication. The animals were anesthetized with a prescribed dose of IsoFlo^®^ (isoflurane, USP, Abbott Laboratories, North Chicago, IL, USA) coupled with oxygen (Oxygen USP, A-L Compressed Gases Inc., Spokane, WA, USA) and periodically monitored by respiration rate during the surgery. Once anesthetized, the animals were shaved around the implantation area and cleaned thrice with alternating chlorhexidine and isopropyl alcohol scrubs. As a numbing agent, 0.3 ml of 0.5% Lidocaine HCL (without epinephrine), was administered subcutaneously on each leg near the incision area. An incision was made on the lateral side above the distal femoral condyle, and a unicortical defect of 2.5 mm diameter was made on the lateral epicondyle using the gradually increasing diameter of drill bits at increments of 0.5 mm. The defect site was rinsed with saline to prevent thermal necrosis and remove residual bone fragments, and the implant was placed in the defect. The fascia over the incision, followed by the skin, was then sutured using undyed braided coated MONOCRYL-polyglactin 910 (Ethicon Inc., Somerville, NJ, USA) outer skin was stapled using sterile surgical staples. An anti-inflammatory analgesic, meloxicam (0.2 mg·kg^−1^), and lactated Ringer’s solution-(3 ml) for rehydration were administered post-surgery subcutaneously, and the animals were monitored until they regained consciousness. Postoperative care was carried out for 3 d, with buprenorphine administration every 12 h and meloxicam every 24 h. After 6 weeks, the rats were euthanized by carbon dioxide overdose, followed by thoracic puncture as a secondary measure. The harvested metal-bone explants were fixed in 10% neutral buffered formalin for at least 72 h for tissue infiltration. The Institutional Animal Care and Use Committee of Washington State University (Pullman, WA) approved protocol was followed to perform the experimental and surgical procedure.

#### 2.5.2. Histological analysis.

After fixing the explants in 10% neutral buffered formalin for 72 h, serial dehydration was carried out in ethanol and embedded in polymethyl methacrylate [15]. They were then sliced into thin sections along the longitudinal direction of the metal implantation and the surrounding bone using a Exakt^™^ saw, ground, and mounted on glass slides. Sanderson’s Rapid Bone Staining (SRBS) and Hematoxylin and eosin (H&E) staining were carried out on separate tissue sections for each composition for histological analyses. These stained bone sections were then observed under a Keyence digital microscope (Model VHX-7000, Itasca, IL), exploiting the microscope’s multi-lighting and 3D-depth composition features to observe osteoid and trabecular bone formation at the bone-material interface.

#### 2.5.3. Histomorphometry analysis.

The stained sections were imaged under a Keyence digital microscope (Model VHX-7000, Itasca, IL), and histomorphometry analysis was carried out using the microscope’s multi-lighting and 3D-depth composition features, which allowed for accurate spherical rendering and detailed imaging of the histological features to the slide thickness. SRBS-stained slides were observed for osteoid and trabecular bone. H&E-stained sections were analyzed for any visible markers of the inflammatory response [15]. Quantitative osseointegration at the bone-implant interface was inspected based on modified scoring criteria per ISO 10993:6 (2016) Annex E.2 [23] (table 1). The modifications in the scoring criteria were decided based on parameters relevant to the scope of this research. The final scoring parameters were trabecular apposition, osteoid at the interface, fibrocartilage presence, inflammation (from H&E-stained sections), fibrosis, and tissue ingrowth into the implant. Out of these six parameters, the first three were quantified as the average area fraction of each parameter calculated using Trainable Weka Segmentation in ImageJ [24–26] over 7 different sections under a high-power field (×1000) around the bone-to-implant contact (BIC) region. Individual sections were further divided into 4 segments for the Random Forest Algorithm. The other four parameters were qualitatively evaluated from low-power field images of the tissue sections (supplemental figure S1).

**Table 1.** Modified scoring for quantitative osseointegration at the bone-implant interface following criteria per ISO 10993:6 (2016) Annex E.2.

	Score				
Parameter	0	1	2	3	4
Trabecular apposition	Absent	Minimal, 1%–25%	Mild, 26%–50%	Moderate, 51%–75%	Marked, 76%–100%
Fibrocartilage presence	Absent	Minimal, 1%–25%	Mild, 26%–50%	Moderate, 51%–75%	Marked, 76%–100%
Osteoid at interface	No bone or osteogenic islands	Osteoid	Mostly woven bone	A mix of woven and lamellar bone	Mostly lamellar bone
Fibrosis	Absent	Narrow band	Moderately thick band	Thick band	Extensive band
Tissue ingrowth into the device	Absent	Minimal	Mild	Moderate	Marked

### 2.6. *In vitro* bacterial study

Bacterial culture was carried out for CpTi, Ti6Al4V, Ti3Al2V, Ti3Al2V–2Cu, and Ti3Al2V–3Cu using two relevant bacterial strains: *P. aeruginosa* (gram-negative) for 36 h and *S. aureus* (gram-positive) for 24 and 48 h. Both strains of bacteria are relatively common in post-surgical orthopedic infections. Freeze-dried *P. aeruginosa* (Carolina Biological, NC) and *S. aureus* were rehydrated using rehydration media. Subsequently, dilutions in nutrient broth were made for 0.5 McFarland standard optical density measurement to identify the correct dilution of 1.5 x 10^8^ CFU·ml^−1^. Disc samples were sterilized and studied in triplicate for agar plate colony count and duplicates for SEM characterization. Samples were placed in separate wells in 24 well-plate, and 10^6^ CFU of bacterial colonies were seeded on the autoclaved polished surface augmented with 2 ml of nutrient broth per well. After respective time points, bacterial cells were scraped from the surface of the 3 out of 5 samples using cell scrapers, mixed in 2 ml 0.1 M phosphate buffer saline, and serially diluted to approximately contain colonies between 30 and 300 CFU in 1 *μ*l solution. 1 *μ*l of the respective solutions were streaked on a Cetrimide agar plate (Pseudosel agar, Fisher Scientific, NH) for *P. aeruginosa* and tryptic soy agar plates for *S. aureus*. Bacterial colonies on agar plates were counted after 24 h of incubation at 37 °C, and the antibacterial efficacy was evaluated as a function of bacterial colonies on individual material compositions as

\begin{equation*}N = C \times d \times 1000/l\end{equation*}

\begin{equation*}R = \left( {{N_{{\text{control}}}}\, - \,{N_{{\text{material}}}}} \right){{/}}{N_{{\text{control}}}}\times 100\%,\end{equation*}

where *N* is the calculated number of bacterial colonies observed, *C* is the average colony count on a plate, *d* is the dilution factor, and *l* is the volume of bacterial suspension on the sample. To study the bacterial cell morphology, SEM samples were preserved in 2% glutaraldehyde and 2% paraformaldehyde in 0.1 M phosphate buffer overnight, followed by dehydration, gold coating, and imaging was carried out on a SEM (Apreo, Thermo Scientific, MA, USA).

## 3. Results

Our work first aims to evaluate the impact of the chemistry and structural design of 3D-printed Ti3Al2V alloys on their mechanical and biological performance compared to CpTi and Ti6Al4V. Bulk porosity for as-printed dense and porous structures was evaluated separately using the same structures used for compression testing vis-à-vis Ti6Al4V and Ti3Al2V. For Ti6Al4V, the % bulk porosities were measured to be 3.7, 14.8, and 35.8, denoted as Ti6Al4V-D, Ti6Al4V-P20, and Ti6Al4V-P40, respectively. Porosities for Ti3Al2V were 3.8%, 19.2%, and 42.9% and were denoted as Ti3Al2V-D, Ti3Al2V-P20, and Ti3Al2V-P40, respectively. The 3.6% and 3.8% porosities for Ti6Al4V-D and Ti3Al2V-D are residual. Although optimized processing parameters were used for all compositions, minor variations in porosities were observed due to a change in chemistry and related variations in laser absorptivity during processing.

### 3.1. Phase analysis, hardness, and microstructure

**Figure 2.**
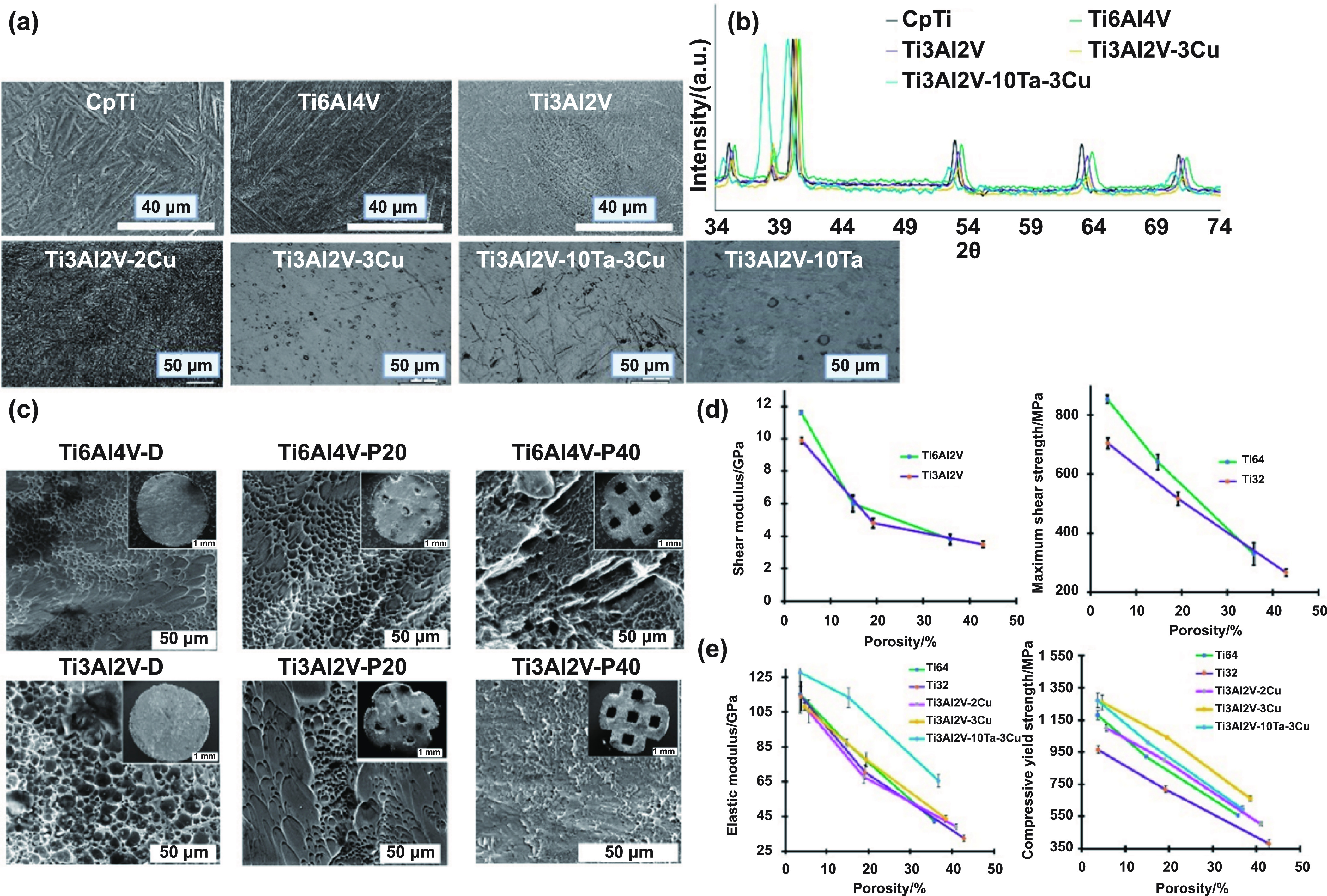
Microstructure, phase analysis, compressive elastic modulus, yield strength, shear modulus, strength, and fracture surface results for the fabricated samples. (a) Microstructure of the samples cut along the build direction showing acicular *α*′ martensitic needles Ti3Al2V, Ti3Al2V–2Cu, and typical *α* and *β* microstructures for CpTi and Ti6Al4V, respectively. However, the needle-like acicular structure shifts to a smoother lamellar microstructure for Ti3Al2V–3Cu, Ti3Al2V–10Ta and Ti3Al2V–10Ta–3Cu compositions; (b) phase analysis from x-ray diffraction; peak shifts observed for Ti6Al4V and Ti3Al2V compared to CpTi due to the addition of alloying elements Al and V. Higher peak shift observed for Ti6Al4V compared to Ti3Al2V. The intensity of all *α*′-Ti peaks except (002) was highest for CpTi due to the introduction of *β*-phase stabilizer V in Ti6Al4V and Ti3Al2V, resulting in reduced amounts of *α*′-Ti. Peak intensity for (002) *α*′-Ti, however, was highest for Ti6Al4V, followed by Ti3Al2V, and lowest for CpTi since this coincides with an enhanced stabilized (011) *β*-Ti peak. A right-shifted peak for Ti3Al2V–3Cu and a left-shifted peak for Ti3Al2V–10Ta–3Cu were observed due to Cu acting as a *β*-Ti stabilizer, while Ta addition results in supersaturated bcc Ti–Ta solid solution, respectively; (c) SEM of fracture surface at the porous-dense interface from the shear test for Ti6Al4V-D, Ti6Al4V-P20, Ti6Al4V-P40, Ti3Al2V-D, Ti3Al2V-P20, and Ti3Al2V-P40. Dimple features were observed at the fracture interface for all compositions, indicating ductile fracture; dimples were more prominent in Ti3Al2V compared to Ti6Al4V, indicating a desired higher ductile behavior for Ti3Al2V before failure; (d) shear modulus and maximum shear strength for porous-dense interface for Ti6Al4V and Ti3Al2V; (e) elastic modulus and compressive yield strength evaluated from the raw compressive stress–strain data plotted against their measured porosities. The effect of reduced Al and V in Ti6Al4V, i.e. Ti3Al2V, showed no prominent effect in elastic modulus compared to Ti6Al4V. However, compressive yield strength decreased for Ti3Al2V for both dense and porous structures. Adding 3% Cu increased the compressive yield strength compared to Ti3Al2V-2Cu; however, no strength increase is seen due to 10 wt.% Ta addition. Shear modulus and maximum shear strength were evaluated by shearing the structures at the porous-dense interface using a single-shear test device plotted against their measured porosities. Reduced amounts of Al and V in Ti6Al4V, i.e. Ti3Al2V, had a noticeable reduction in both the shear modulus and the maximum shear strength endured at the porous-dense interface.

SEM micrographs on etched surfaces for all the compositions, figure 2(a), displayed highly anisotropic acicular *α*′ martensitic needles for Ti3Al2V similar to CpTi and Ti6Al4V. The acicular needle-like characteristic phase is present even for Ti3Al2V–2Cu composition; however, adding more Cu content and 10 wt.% Ta resulted in the refinement of grains and a change in microstructure from needle-like to more lamellar, which is consistent with the solid-solution microstructure of Ti–Ta alloys [15]. The most prominent XRD peaks observed for all compositions are for *α*′-Ti, figure 2(b) and supplemental figure S2. No prominent *β*-Ti peaks were observed, similar to previously reported results [27]. We observe a shift in the peaks for Ti6Al4V and Ti3Al2V towards the right compared to CpTi peaks due to alloying elements Al and V, making them an *α* + *β*-Ti alloy as opposed to *α*′-Ti in CpTi. This shift is higher for Ti6Al4V compared to Ti3Al2V due to the presence of a higher amount of *β*-Ti. Comparing the peak intensities, for all the peaks except *α*′-Ti (002), the highest intensity was observed for CpTi, followed by Ti3Al2V, and the lowest for Ti6Al4V. Although the difference in the peak intensities is insignificant, the peaks for CpTi display the highest peak intensity values due to the higher *α*′-Ti presence. Comparing Ti6Al4V and Ti3Al2V, due to lower contents of alloying elements of Al and V in the latter, the amount of *α*-Ti is higher in Ti3Al2V than that of Ti6Al4V. Observed in the *α*′-Ti (002) the trend in the peak intensities is reversed. The highest peak is observed for Ti6Al4V, followed by Ti3Al2V, and the lowest for CpTi. We hypothesize that this is a combination peak intensity of the *β*-Ti peak (011) and the *α*-Ti peak for (002), as also observed [28]. For Ti3Al2V-3Cu, all the *α*′ peaks, except *α*′ (002) and *α*′ (101), observe a rightward shift and lower peak intensities for *α*′-Ti in comparison to those Ti3Al2V indicating increased *β*-Ti phase since Cu is a *β*-Ti stabilizer. For *α*′ (002) peak, we observe a higher intensity peak for Ti3Al2V–3Cu than that for Ti3Al2V. As mentioned, this peak coincides with the *β*-Ti (110) peak, indicative of a higher *β*-Ti phase in Ti3Al2V–3Cu. No Cu peaks were observed. For Ti3Al2V–10Ta–3Cu, we observed *α*″-Ti do to rapidly quenched supersaturated bcc-Ti–Ta solid solution [15]. The *α*″ (002) coinciding with the peak has a very high intensity in the *β* (110) peak, indicating a high amount of *β*-Ti formation since Ta is also a *β*-Ti stabilizer. Vickers microhardness values for CpTi, Ti6Al4V, and Ti3Al2V were (264 ± 11), (386 ± 15), and (303 ± 12) HV_0.2_, respectively. Ti3Al2V followed the highest hardness value of Ti6Al4V due to the latter’s higher content of alloying elements. Hardness values for Ti3Al2V–2Cu and −3Cu were (353 ± 17) and (382 ± 26) HV_0.2_, respectively. The hardness enhancement is due to the formation of intermetallic Ti_2_Cu and solid solution strengthening by Cu solute atoms; the higher the amount of Cu, the higher the hardness value observed (2 vs. 3 wt.% Cu). The hardness value for Ti3Al2V–10Ta–3Cu was (342 ± 8) HV_0.2,_ indicating Ta did not increase hardness (supplemental figure S3).

### 3.2. Mechanical strength characterization: compression, shear, and fatigue

Metallic implants at load-bearing sites are constantly under multi-axial loading *in vivo*. It becomes essential to understand the mechanical behavior of such implants under varying loading conditions to minimize the possibility of implant failures. Dense and porous samples for all compositions were subjected to compression loading, while dense-porous interface samples for Ti6Al4V and Ti3Al2V were subjected to shear loading, and only dense samples were subjected to fatigue loading. AM-processed Ti6Al4V was used as a positive control.

#### 3.2.1. Shear behavior.

The weakest point lies in the porous-dense interface material design for porous coatings on the bulk implant. For Ti3Al2V to be a more versatile material, it is essential to evaluate the shear characteristics at this porous-dense interface to prevent coating failures in such coating applications [29]. SEM micrographs at the porous-dense failure interface, figure 2(c), reveal ductile dimples for both compositions, indicating a desirable ductile behavior. It can be corroborated from the raw shear stress*–*strain plots, supplemental figure S4(a), Ti3Al2V-D shows higher ductility than Ti6Al4V-D before failure, and the same for Ti3Al2V-P20 and Ti6Al4V-P20. Even with increased porosity at the porous-dense interface, the ductility was comparable for Ti3Al2V-P40 and Ti6Al4V-P40. Shear stress*–*strain plots from tests are presented in supplemental figure S4(a). Shear modulus and maximum shear strength are plotted against their porosities at the porous-dense interface in figure 2(d). It should be noted that the nature of the shear test performed is tensile rather than torsional. Reduced shear modulus and strength were observed at the porous-dense interface from Ti6Al4V to Ti3Al2V. Shear modulus for dense structures Ti6Al4V-D and Ti3Al2V-D were 11.6 and 9.9 GPa, i.e. reduced Al and V content from Ti6Al4V-D to Ti3Al2V-D led to a 15% reduction in shear modulus. Thus, it was observed that the change in shear modulus was a function of the compositions. Maximum shear strength endured by Ti6Al4V-D and Ti3Al2V-D were 853 and 705 MPa; a 17% reduction in maximum shear strength was observed with a reduction in Al and V. For the P20 group, shear modulus for Ti6Al4V-P20 (6 GPa, 14.8% porosity) was higher than that for Ti3Al2V-P20 (4.8 GPa, 19.2% porosity), and the shear strengths were 640 and 516 MPa, respectively; 20% shear modulus reduction and 19% shear strength reduction from Ti6Al4V-P20 to Ti3Al2V-P20 was observed, given that Ti3Al2V-P20 had a 4.4% higher porosity. For the P40 group, the shear modulus difference between Ti6Al4V-P40 (3.8 GPa, 35.8% porosity) and Ti3Al2V-P40 (3.5 GPa, 42.9% porosity) decreased; only an 8% reduction in shear modulus was observed from Ti6Al4V-P40 to Ti3Al2V-P40, given that Ti3Al2V-P40 had 7.1% higher porosity. Comparing shear strength values for Ti6Al4V-P40 (330 MPa, 35.8% porosity) and Ti3Al2V-P40 (267 MPa, 42.9% porosity), a reduction of 19% in shear strength was observed from Ti6Al4V to Ti3Al2V, given that Ti3Al2V-P40 had 7.1% higher porosity than Ti6Al4V-P40. It can be seen from figure 2(d) that with an increase in porosity at the maximum strength, the reduction was greater for Ti6Al4V compared to Ti3Al2V.

#### 3.2.2. Compressive behavior.

It is desired for an implant material to demonstrate an elastic modulus closer to that of the natural bone (∼5–30 GPa), with high yield strength. This reduction in elastic modulus is achieved by introducing controlled porosity in metallic implants. Elastic modulus and compressive yield strength values for dense and porous Ti6Al4V and Ti3Al2V against their evaluated porosities are plotted in figure 2(e). CpTi and Ti6Al4V display similar elastic modulus values, ∼110 and ∼114 GPa, respectively. Since Ti3Al2V is a mixture of the two compositions, it is not expected that Ti3Al2V’s elastic modulus will vary compared to Ti6Al4V. The variation in elastic modulus between Ti6Al4V and Ti3Al2V can be attributed primarily to the porosity difference. The elastic modulus for Ti6Al4V-D and Ti3Al2V-D were similar, 115.3 and 114.1 GPa, respectively. For the P20 structures, Ti6Al4V-P20 (porosity 14.8%) showed an elastic modulus of 87.2 GPa, and that for Ti3Al2V-P20 (19.2%) was 70.3 GPa. Similarly, for P40 structures, the modulus for Ti6Al4V-P40 (porosity 35.8%) was 42.3 GPa, and that for Ti3Al2V-P40 (porosity 42.3%) was 32.5 GPa. Dense Ti3Al2V–3Cu displayed a modulus of 108.2 ± 2.1 GPa, and when Ta is added to Ti3Al2V–3Cu to form Ti3Al2V–10Ta–3Cu, the elastic modulus was observed to be 144.3 ± 10.2 GPa. Cu and Ta are *β*-Ti phase stabilizers, but the high cooling rates of AM prevented the formation of the *β*-Ti phase, resulting in no effect of lower modulus value by Cu addition (pure Cu modulus ∼130 GPa) and modulus enhancement due to Ta addition since pure Ta has a modulus of ∼185 GPa. As stated before, minor porosity variations happened due to compositional variations, even with optimized processing parameters.

As opposed to the high compressive yield strength of Ti6Al4V ∼1100 MPa, CpTi shows a yield strength as low as 432 MPa [30]. This enhanced strength in Ti6Al4V is due to the addition of Al and V solute atoms inducing the formation of a high-strength-low modulus *β*-Ti phase in Ti6Al4V. The variation in compressive yield strength can be attributed to the composition and porosity. An 18% reduction in strength from Ti6Al4V-D (1181 MPa) to Ti3Al2V-D (965 MPa) was observed when Al and V were reduced. From Ti6Al4V-P20 (922 MPa) to Ti3Al2V-P20 (719 MPa), a 22% reduction in compressive yield strength, with 4.4% higher measured porosity for Ti3Al2V-P20 than Ti6Al4V-P20 is observed. Similarly, Ti6Al4V-P40 (557 MPa) to Ti3Al2VP40 (382 MPa), with 7.1% higher porosity for Ti3Al2V-P40, reduced compressive yield strength by 31%. At the same time, the strength observed for Ti3Al2V-P40 (382 MPa, 42.9% porosity) is comparable to that of dense CpTi (∼350 MPa). For Ti3Al2V–10Ta–3Cu, the compressive yield strength increased to (1255 ± 51) MPa. This enhancement in strength is due to the formation of Ti_2_Cu intermetallic formation in Ti3Al2V–10Ta–3Cu alloy and the solid solution strengthening effect due to Cu. Raw stress vs. strain plots for each porosity and composition are reported in supplemental figures S4(b)–(d).

#### 3.2.3. Fatigue behavior.

AM-processed dense fatigue samples of Ti6Al4V, Ti3Al2V, and Ti3Al2V–10Ta–3Cu at a 90° build angle were tested under fatigue loading as that is the weakest build direction due to being parallel to the loading direction. The fatigue specimens were turned to the final shape to minimize AM-generated surface roughness at the gauge length. Samples were then heat treated at 400 °C for 1 h and then furnace cooled to reduce residual stresses. After removal from the furnace, the sample is polished using emery cloth ranging from 400 to 600 grits until no defects are visible on the surface. Samples were tested to determine at what stress amplitude samples can survive at least 10 million cycles without failure. Ti6Al4V and Ti3Al2V samples survived 10 million cycles at 21% of their respective compressive yield strength. Anisotropy is a critical factor in additively manufactured structures. Ti3Al2V structures at 0° build angle demonstrated almost two times higher fatigue endurance limit than Ti3Al2V at 90° [31]. With a further increase in stress amplitude, samples failed at lower cycles. For the Ti3Al2V–10Ta–3Cu samples, no failure up to 10 million cycles was accomplished at 19% of the compressive yield strength. Our initial fatigue test results indicate that lowering Al and V in Ti and adding Ta and Cu do not degrade the excellent fatigue response of these alloys.

### 3.3. Tribological study

**Figure 3.**
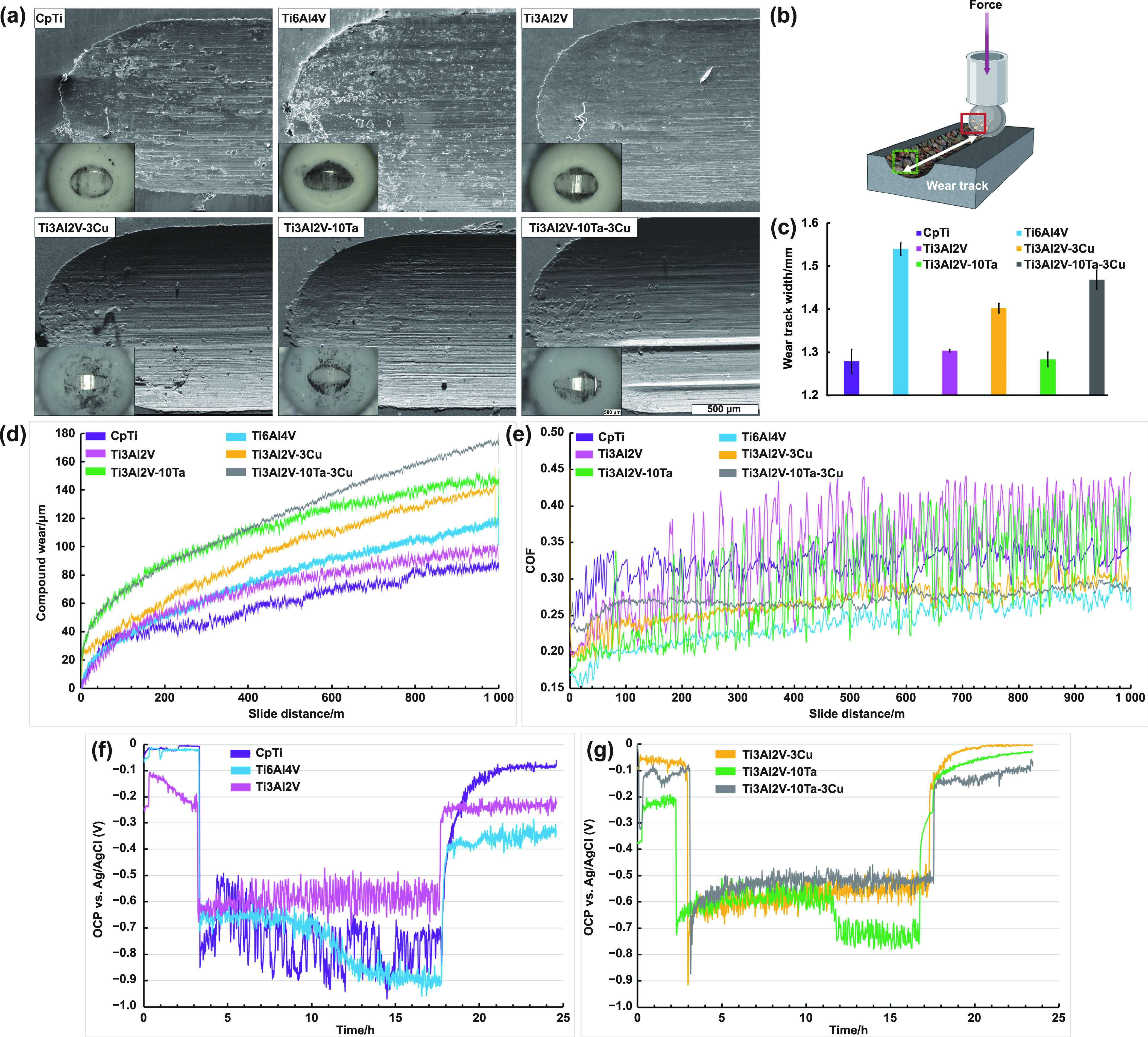
Pictograms of the wear track and wear ball for (a) CpTi, Ti6Al4V, Ti3Al2V, Ti3Al2V–10Ta, Ti3Al2V–3Cu, and Ti3Al2V–10Ta–3Cu. Scale bar represents 500 *μ*m and is uniform or all wear track images; (b) tribology testing displaying the wear track’s formation, and (c) measured wear track width. Statistical analyses were done using one-way ANOVA for *α* = 0.05. Tukey–Kramer simulations were done for pairwise comparisons of wear track width with a *P*-value <0.05 considered significantly different. During Tribological testing, acquisition of the (d) compound wear, (e) coefficient of friction (COF), and (f) and (g) OCP measurement curves.

Tribological testing and characterization are essential to any material intended for use in physiological load-bearing sites. Proper interpretation of the physical wear phenomena must be evaluated from the CW curve, COF, and worn surface imaging of the tested sample and the counterwear material; this is needed to determine the wear-induced degradation and material deformation thoroughly. Additionally, an investigation into the electrochemical passive nature of the material during tribological testing should be done. Such testing requires a data logging unit, known as a potentiostat, a WE, a RE, and an electrically conductive media or an electrolyte. The results of such tribo-corrosive testing allow for quantification and further understanding of the material’s chemical behavior or evolution during tribological testing; chemical changes usually occur within the passivation, de-passivation, and re-passivation domains. In the presence of physiological or simulated body fluid, the testing can be *in* *vitro* in nature. The tribo-biocorrosive results for these alloys are displayed in figure 3.

#### 3.3.1. Wear behavior.

Electron micrographs of the wear track surface and optical images of the counter wear ball were attained and are displayed in figure 3(a). A rendering of the physical interaction between the wear ball and the sample is displayed in figure 3(b). CpTi displayed the most gouging, plastic deformation, material removal, transfer, and subsequent material deposition on the wear track itself; Ti6Al4V was comparable in appearance, while the Ti3Al2V wear track was smoother. Across all Ta and Cu compositions, a decrease in the material adhesive transfer to the worn ball was observed, implying a suppression in the wear mode during tribological testing. When measuring the wear scar width on the ZrO_2_ wear balls, the values were in ascending order: 387 *μ*m, 423 *μ*m, 453 *μ*m, 519 *μ*m, 564 *μ*m, and 657 *μ*m for Ti3Al2V–10Ta–3Cu, Ti3Al2V–10Ta, Ti3Al2V–3Cu, Ti3Al2V, Ti6Al4V and finally CpTi, respectively. The measured values are proportional to the chord length associated with the arc length of the radial surface of interaction between the tested sample and the ZrO_2_ wear ball, i.e. the curvature of the wear track trough. A longer linear measurement is indicative of increased wear on the ZrO_2_. In the current study, the visible wear on the ZrO_2_ increases in the order of Ti3Al2V–10Ta–3Cu, Ti3Al2V–10Ta, Ti3Al2V–3Cu, Ti3Al2V, Ti6Al4V and finally CpTi, therefore Ti3Al2V–10Ta–3Cu displays the least and CpTi displays the most amount of wear on the ZrO_2_ counter wear ball. When measuring wear track width, Ti6Al4V displayed the greatest width at ∼1.55 mm, while CpTi, Ti3Al2V, and Ti3Al2V–10Ta had comparable wear track widths at ∼1.3 mm, as displayed in figure 3(c). Final CW, as displayed in figure 3(d), for the tested compositions was ∼175 *μ*m, ∼147 *μ*m, ∼143 *μ*m, ∼117 *μ*m, ∼98 *μ*m and ∼88 *μ*m for Ti3Al2V–10Ta–3Cu, Ti3Al2V–10Ta, Ti3Al2V–3Cu, Ti6Al4V, Ti3Al2V and CpTi, respectively. Ti3Al2V–10Ta and Ti3Al2V–10Ta–3Cu displayed the greatest running-in wear from 0 to 40 m. Among all 6 compositions, Ti3Al2V–3Cu, Ti3Al2V–10Ta–3Cu, and Ti6Al4V exhibited the lowest COF, roughly 0.25 at 1000 m, as shown in figure 3(e). However, Ti6Al4V remained with a constant positive slope while Ti3Al2V–10Ta–3Cu exhibited a steady-state near a moving average of zero slope in the COF.

#### 3.3.2. OCPs.

The acquisition of the OCP before and during tribological testing results in the curves displayed in figures 3(f) and (g). The idle OCP (*E*_Idle_) is first attained when the sample is submersed into the media and is allowed to reach equilibrium potential. Once tribological testing commences, the OCP drops to anodic potentials when compared to *E*_Idle_. The OCP attained at this instance is referred to as the wear OCP (*E*_Wear_) and can dynamically change depending on a change in surface chemistry during tribological testing. Another essential quantification derived from a tribologically attained OCP curve is the Δ*E* potential ($|$ *E*_Wear_ − *E*_Idle_ $|$_ =_ Δ*E*)—the magnitude of the change in OCP from idle to wear conditions. OCP acquisition before tribological testing revealed that Ti6Al4V and CpTi initially demonstrated the most cathodic (positive) potential under idle conditions, with Ti3Al2V exhibiting the most anodic (negative), figure 3(f). Upon tribological testing, an anodic shift across all compositions was observed, with Ti6Al4V exhibiting the most anodic shift and Ti3Al2V–10Ta–3Cu exhibiting the most cathodic, as displayed in figure 3(g). As wear testing progressed, both CpTi and Ti6Al4V shifted to a more anodic potential relative to the start of the wear regime and when compared to Ti3Al2V, which shifted to a slightly more cathodic potential stabilizing to ∼−0.6 V. Ti3Al2V–3Cu and Ti3Al2V–10Ta–3Cu shifted to a more cathodic potential comparable to CpTi, Ti6Al4V and Ti3Al2V–10Ta. Upon unloading Ti3Al2V–3Cu, Ti3Al2V–10Ta, and Ti3Al2V–10Ta–3Cu re-passivated to more positive OCP values when compared to CpTi, Ti6Al4V, and Ti3Al2V. It was observed that the presence of Cu in the Ti3Al2V matrix allowed for a cathodic shift in OCP within the depassivation (tribological testing) and re-passivation domain.

### 3.4. Histological analysis—bone-implant interface

**Figure 4.**
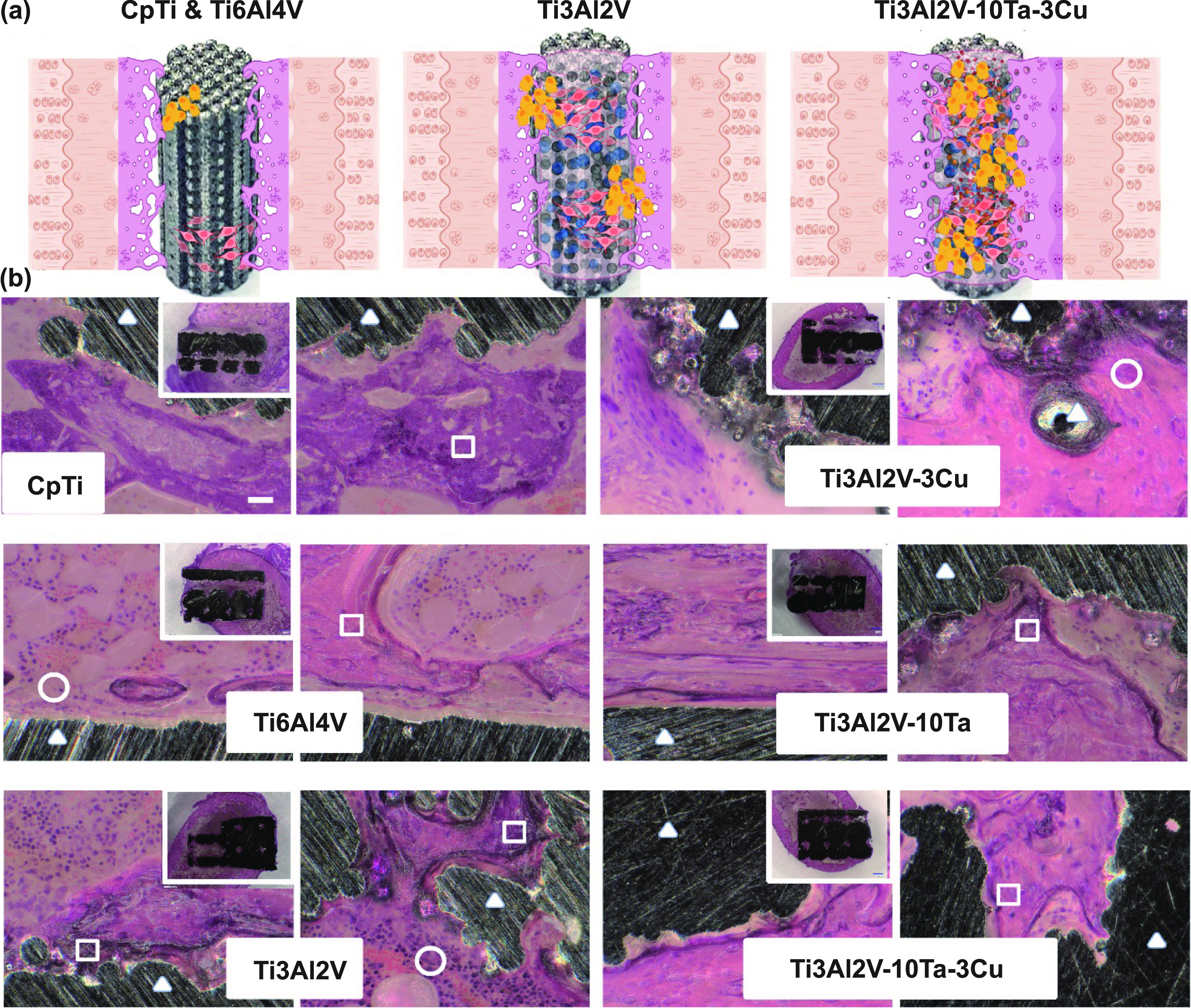
*In vivo* Hematoxylin & Eosin (H&E)-stained histology images. (a) Schematic of *in vivo* native bone integration into surgically placed metallic implants showing a variation of osseointegration ability as a function of material properties. (b) Histology micrographs of tissue cross-sections at the bone-implant interface stained with H&E for porous implants. The black area is the implant (denoted with triangles), with the dark pink area representing the trabecular bone formed (square), lighter pink areas with a smooth appearance is fibrocartilage, the light pink area with rounded cell nuclei is osteoid tissue, and purple dots in osteoid tissue represents the osteoblast cells in the bone integration front (circle). The pore cross-section shown is not the same for all compositions. Infiltration of newly formed trabecular bone into the pores is observed for Ti3Al2V-P along with good amounts of matured bone on the implant’s outer surface compared to CpTi (gaps at the BIC) and Ti6Al4V. No evidence of inflammatory response was noticed for any composition. The scale bar in the first image represents 25 *μ*m and is uniform for all images.

**Figure 5.**
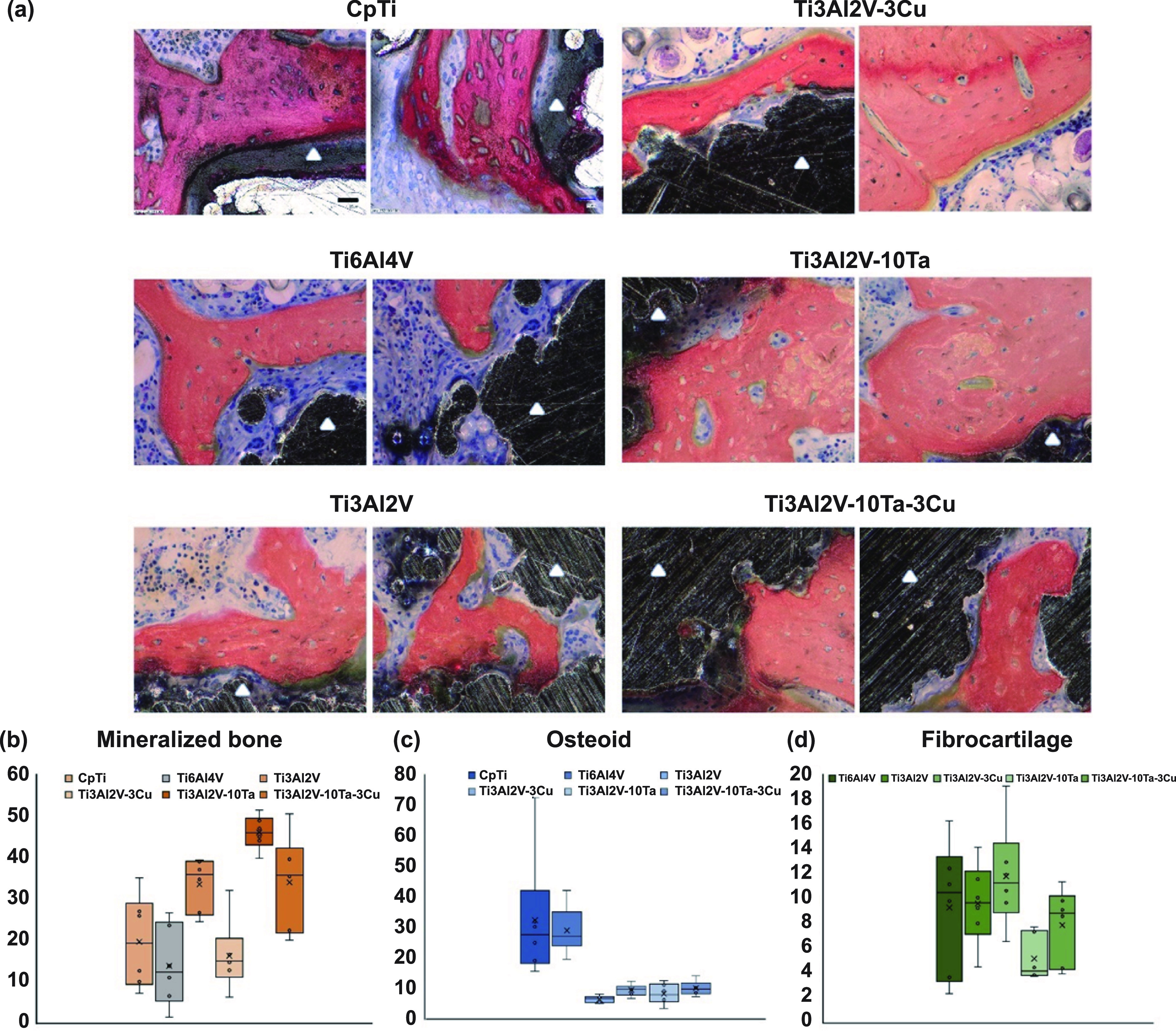
Quantification of bone formation and maturation (a) Sanderson’s Rapid Bone-Stained micrographs (SRBS). Reddish/orange areas represent matured trabecular bone, while darker and lighter blue areas represent osteoid tissue or a combination of fibrocartilage with osteoid tissue, respectively. Dark blue rounded dots are osteoblasts recruited in the newly formed osteoid tissue at the bone integration front. Some areas of a lighter blue with more elongated cells represent chondrocyte cells in the fibrocartilage. Ti3Al2V clearly shows bone apposition and osseointegration at the BIC compared to clear gaps for CpTi and Ti6Al4V. The presence of well-embedded osteocytes in the trabecular area for Ti6Al4V composition at 4 weeks post-implantation suggests older bone that could be a residual continuation from the outer cortical area. Adding Cu to Ti3Al2V composition does not elicit any inflammation or neoplasia, suggesting non-toxicity; however, there is evidence of the delayed onset of osseointegration compared to Ti3Al2V composition alone. Positive control Ti3Al2V–10Ta shows very well-integrated trabecular bone at the BIC with trabecular bone width higher than the rest of the compositions. Interestingly, adding 10 wt.% Ta to Ti3Al2V–3Cu reverses the delayed onset of osseointegration and shows the overall best performance among all six compositions. Graphs present a quantitative analysis of the observations made from SRBS-stained histology sections for (b) mineralized bone, (c) osteoid, and (d) fibrocartilage based on ImageJ Trainable Weka Segmentation. Statistical information from Tukey–Kramer pairwise comparisons is presented in supplemental tables ST1-ST3. The scale bar in the first image represents 25 *μ*m and is uniform for all images.

*In vivo* rat model with CpTi as the positive and Ti6Al4V as the negative control was studied with dense and porous implants. Figure 4(a) represents a schematic of an *in vivo* bone integration with the surgically placed implant. Figure 4(b) and supplemental figure S5 show H&E-stained bone sections for porous and dense implants, respectively; dense implants were primarily considered an *in vivo* control for comparison. The H&E-stained bone sections for porous implants presented in figure 4(b) show pore cross-sections from different layers for each composition. The H&E staining for the bone-implant sections was primarily evaluated for *in vivo* inflammatory response towards Ti3Al2V, Ti3Al2V–3Cu, and Ti3Al2V–10Ta–3Cu chemical makeup as an implant material. None of the three material compositions exhibited inflammatory markers or responses, including necrosis and neoplasia. Even though H&E histology micrographs are represented in varying shades of pink/purple, visible demarcations are present for all compositions, which aids in determining their relative biological responses [32]. Figure 5(a) and supplemental figure S6 show SRBS-stained bone sections for porous and dense implants, respectively. The SRBS histology micrographs for dense and porous implants show qualitative evidence of new bone or osteoid formation, osteoblast recruitment, and bone maturation or trabecular bone formation in the region of interest across all compositions except for Ti6Al4V. However, detailed analysis suggests gaps at the BIC surface with multiple focal fibrocartilaginous areas at BIC for CpTi. Markedly, there was no significant bone ingrowth into the pores in CpTi. Some areas show interwoven lamellar bone into the fibrocartilage in Ti3Al2V. The lighter blue stain indicates the onset of fibrocartilage with more flattened and organized elongated cell rows. In comparison, Ti6Al4V shows almost 90% of the interface area covered with fibrous tissue (dark blue), including pore infiltration with isolated areas of osteoid presence. Some areas show old/matured cortical bone segments from surgical procedures. Fibrocartilage presence at a distance from the implant shows inferior osseointegration affinity towards the implant material. On the other hand,

Ti3Al2V displayed no gaps at the BIC with very well-apposed osteoid tissue (reddish orange) and continuing osteoblast recruitment. Bone tissue infiltration takes precedence over fibrocartilage into the porous channels of the bulk implant.

Ti3Al2V–3Cu alloys show similar characteristics of bone remodeling to CpTi implants with osseous tissue at the BIC and interwoven lamellar bone into the implant surface. The fibrocartilage that runs through the porous channel has focally embedded new bone tissue. Ti3Al2V–10Ta shows enhanced bone ingrowth into the implant’s pores and osteoid lining fronts, suggesting continued bone remodeling. However, the fibrocartilaginous presence in Ti3Al2V–3Cu alloys is shown to be restored to the bony formation and new osteoid tissue inside the pores of the implant in Ti3Al2V–10Ta–3Cu compositions with the addition of 10 wt.% Ta.

Quantitative histomorphometry (table 2 and figures 5(b)–(d)) provides a clearer idea about the remodeling process undergone for all six compositions at 6 weeks post-implantation. Comparing the box plots for mineralized bone and osteoid presence at the BIC (figures 5(b) and (c)), there is a significant change in bone maturation between CpTi, Ti6Al4V, and the rest of the compositions, indicating enhanced biocompatibility and biological response of native tissue towards the implant material. Overall, Ti6Al4V shows the least amount of mineralized bone (14 ± 8), which is not significantly different from that of CpTi (19.7 ± 10), which corresponds to the higher values for osteoid presence at the BIC for these compositions (29 ± 7) and (32.4 ± 19) respectively. However, with changing the chemical composition of the alloy to Ti3Al2V, a significant jump in native tissue response is observed with the fraction of mineralized bone area (34 ± 5) compared to Ti6Al4V. This jump is reflected in the lower area fraction of osteoid in Ti3Al2V (6.7 ± 1). With the addition of 3 wt.% Cu to the modified alloy Ti3Al2V, although we notice a significant change from Ti3Al2V, the area fraction of mineralized bone does not significantly change from that of CpTi. Therefore, it is safe to deduce that bone maturation might be delayed due to Cu addition; biocompatibility is not compromised. Since Ti3Al2V–10Ta (positive control) shows the highest amount of mineralized bone (46 ± 3), adding 10 wt.% Ta to Ti3Al2V–3Cu alloys speeds up the delayed bone remodeling at the same level as Ti3Al2V-10Ta as well as Ti3Al2V alloys owing to non-significant statistical differences.

**Table 2.** Showing scored parameters for bone regeneration around BIC based on scoring criteria mentioned earlier.

	CpTi	Ti6Al4V	Ti3Al2V	Ti3Al2V–3Cu	Ti3Al2V–10Ta	Ti3Al2V–10Ta–3Cu
Trabecular apposition	1	1	2	1	2	2
Fibrocartilage presence	0	1	1	1	1	1
Osteoid at the interface	1	1	3	2	4	3
Inflammation	0	0	0	0	0	0
Fibrosis	0	2	0	1	0	0
Tissue ingrowth into the device	1	0	3	1	4	4

The box-plot for fibrocartilage presence in figure 5(d) depicts a uniform fibrocartilage presence across all the compositions whether visibly at the BIC extended from the lateral edge of the implant exposed to multiple different tissues apart from the osseous phenotype. Such fibrocartilage response is expected at an early timeframe, such as 6 weeks, which then develops into woven bone. However, there is only a significant difference in fibrocartilage presence in Ti3Al2V–3Cu (11.8 ± 4) and Ti3Al2V–10Ta (5.2 ± 2) due to much higher trabecular bone presence for Ti3Al2V–10Ta (46 ± 3) compared to other compositions.

### 3.5. Infection prevention and bacterial resistance

**Figure 6.**
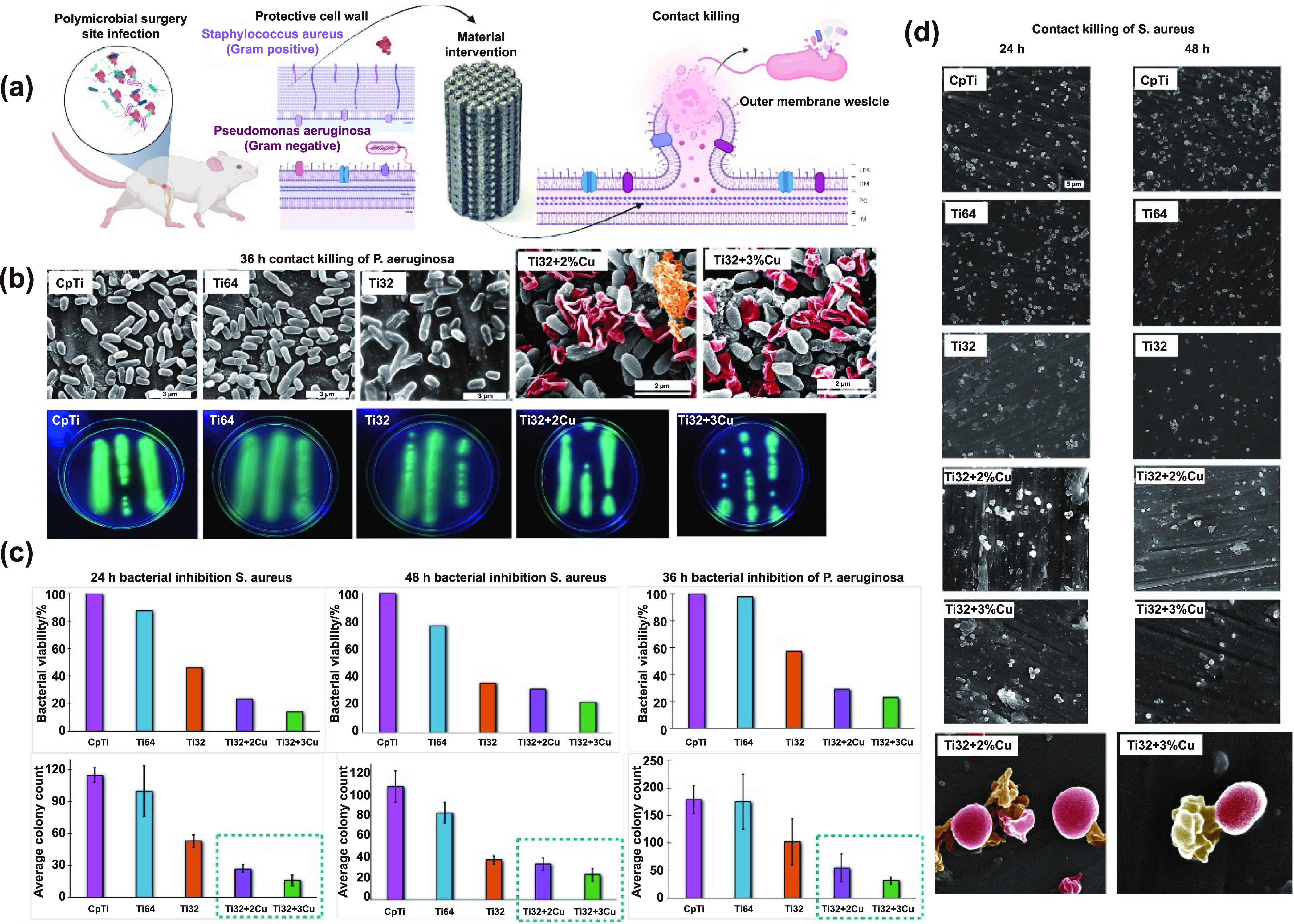
Results from antibacterial study with Pseduomonas aeruginosa and Stapphylococcus aureus bacterial strains. (a) Schematic of intrinsically antibacterial material intervention towards post-surgical secondary infection at the surgery site. Bacterial inhibition characteristics of a material depend on the bacterial type, cell wall structure, and mode of killing. (b) *P. aeruginosa* bacterial study after 36 h of culture; Cetrimide agar plate images showing bacterial colonies across all compositions; SEM images showing the deflated bacterial cell wall-morphology for Ti3Al2V–2Cu and Ti3Al2V–3Cu due to cytoplasmic outflow as a result of toxicity from the Cu towards bacterial cells; (c) bacterial cell viability and bacterial colony count from agar plate counting (*n* = 3), showing decreased bacterial viability for Ti3Al2V, Ti3Al2V–2Cu, and Ti3Al2V–3Cu compared to CpTi and Ti6Al4V. (d) *S. aureus* bacterial cell culture; bacterial cell viability and bacterial cell count from SEM images (*n* = 4) showing decreased bacterial viability for Ti3Al2V, Ti3Al2V–2Cu, and Ti3Al2V–3Cu compared to CpTi and Ti6Al4V with cytoplasmic outflow observed even as early as 24 h.

Both CpTi and Ti6Al4V do not possess inherent bacterial resistance [33, 34]; therefore, we do not expect any antibacterial properties in Ti3Al2V by extension. However, since post-surgical implant site infection is a common denominator in orthopedic material applications (figure 6(a)), examining currently used orthopedic materials is not beyond the scope of this research. As a confirmatory test, bacterial viability across all compositions was tested for 36 h with *P. aeruginosa* (gram-negative) colonies. Cetrimide (Pseudosel agar) is a selective medium to culture this bacterium. SEM micrographs (figure 6(b)) also corroborate a reduction in planktonic *P. aeruginosa* bacterial cells on the surface of Ti3Al2V compared to that of CpTi and Ti6Al4V. However, bacterial inhibition was significantly enhanced after 36 h of culture for Ti3Al2V–2Cu and Ti3Al2V–3Cu compositions, showing deflated bacterial cells from cytoplasmic outflow (in red). *P. aeruginosa* secretes a pigment pyoverdine that emits fluorescent green color under UV light on Cetrimide agar, showing significantly lesser colony formation on agar plates for Ti3Al2V–2Cu and Ti3Al2V–3Cu, figure 6(c). Percent bacterial viability evaluated for all compositions shows the antibacterial efficacy of Ti3Al2V–2Cu and 3Cu alloys, which were 70% and 80% higher than CpTi after 36 h of culture, respectively. The antibacterial efficacy of CpTi and Ti6Al4V was observed to be within the error margins of each other and under 4% since neither showed any antibacterial properties. However, for Ti3Al2V, the reduction in live bacteria on the surface by 57% was a marked and novel identification of material properties for Ti3Al2V alloys.

This data was valuable and crucial for establishing Ti3Al2V–Cu alloys as a potential material for implant applications. Therefore, to confirm the antibacterial efficacy of these alloys, we performed a secondary bacterial culture with a different strain of bacteria *S. aureus* (gram-positive), for 24 and 48 h on all compositions. Bacterial cells were counted in triplicate from SEM micrographs/unit area based on magnification instead of the agar plate method. Percent living bacteria for Ti3Al2V–2Cu and 3Cu compared to CpTi at 24 h of culture were 23% and 14%, respectively. However, at 48 h there was a slight but insignificant increase in the bacterial count for both compositions. We observed a similar result as with the previous bacteria; for Ti3Al2V, the % bacterial viability was significantly lower than CpTi and 46% and 36% after 24 and 48 h of culture, respectively. Even though the antibacterial efficacy of Ti3Al2V is a novel finding, exploring the detailed microbiological mechanism behind such an observation is beyond the scope of this research. However, this observation enhances the scope of Ti3Al2V–Cu alloys for suggested application. According to the SEM micrographs shown in figure 6(d), significantly lower *S. aureus* bacterial cells can be observed in SEM images on the surface of Ti3Al2V compared to that on CpTi and Ti6Al4V for both time points. We observed a ruptured cell wall and disruption of the cell membrane for *S. aureus* bacterium on Ti3Al2V–2Cu and 3Cu. Although we do not know the reason behind this bacterial resistance in Ti3Al2V, further investigation is needed to understand the toxic effect of Ti3Al2V on bacterial cells.

## 4. Discussions

The concept of ‘race to the surface’ was introduced in 1987 [35], describing the antagonistic behavior of host cells and contaminating bacteria to occupy the biomaterial surface [36]. The challenge is weaponizing implants that help osseous tissue win the ‘race’ and cover the biomaterial surface while inhibiting bacterial colonization. Problems with current systemic infection treatment spread over domains like (1) low drug concentration at the infection site, (2) systemic drug exposure and potential toxicity, (3) no control over drug release, and (4) risk of emergence of resistant organisms [37]. In 2020, the Centers for Disease Control and Prevention reported >2.8 million resistant infections yearly. Therefore, there is an unmet need to design implants with intrinsic material properties that can provide bacterial resistance, which is a more reliable way of determining the overall clinical significance of implant materials. Some of the major inclusion criteria for the U.S. Food and Drug Administration’s standard operating procedure (2018), ‘determining whether the presence of an antimicrobial agent raises new questions of safety and effectiveness’ for 510(k) eligibility are (1) incorporation of the antimicrobial agent into the device, (2) the device and the antimicrobial agent must be used at the same anatomical site (this excludes systemic antibiotic drugs), and (3) the device and the agent has the same design characteristics (such as material, geometry, function). This reflects that single-vector research on improving the biocompatibility of a metallic biomaterial is no longer sufficient in determining their eligibility for application. Considerations on all verticals that make an implant comprehensively biocompatible are the need of the hour, which is also the focus of our research.

Considering materials chemistry is one of the primary characteristics that modulate an event like bacterial colonization, alloying Cu with Ti for bacterial contact-killing and eliminating the need for revision surgery has become a growing research focus [14, 38–40]. Although Cu is an essential trace element in the body, higher amounts of Cu (II) released in the physiological environment can cause cytotoxicity and present concerns. The optimum amount of Cu in Ti alloy without any toxic effect is still debatable [14, 41–47]. Table 3 shows the lack of vertical integration of biocompatibility aspects regarding Cu alloys used in metallic implants. Our research employs multi-material additive manufacturing to integrate these verticals by (1) incorporating Cu (2 and 3 wt.%) in Ti as an antimicrobial agent for continued bacterial resistance and help osseous tissue win the ‘race’ to the surface and (2) modifying Ti6Al4V alloys into Ti3Al2V combined with the addition of 10 wt.% Ta to compensate for compromised biocompatibility (if any) from Cu addition and enhance osseointegration.

**Table 3.** Literature summary of available information on Ti–Cu alloys researched as antimicrobial metal implants. The table lists information on fabrication, % Cu metal used, characterizations done, and if steps have been taken to counter any drawbacks from Cu addition, represented as a comparison with our presented research.

Publication	Materials (% Cu addition)	Fabrication	3DP (Yes/No)	Supplementary measures to enhance biocompatibility	Coating strength assessment	Mechanical Assessment of alloy	*In vivo* biocompatibility tests (Yes/No)
Present Comprehensive Study	Ti6Al4V +CpTi + **3 wt.% Cu** + **10 wt.% Ta**	SLM	**Yes**	Reducing Al, V content in Ti6Al4V by adding CpTi (Ti3Al2V)	3DP Ti3Al2V coating on Ti6Al4V via assessing shear modulus	Yes	**Yes**
						(Elaborated in the study)	
						Tribological study	
				Enhancing osseointegration by adding 10% Ta		Electrochemical corrosion assessment	
						Fracture surface assessment	
				Incorporating porosities			
[1] [41]	CpTi +**5 wt.% Cu**	Electrode arc-melting followed by forging	**No**	None	–N/A–	None	**No**
[2] [48]	CpTi +**5 wt.% Cu**	Vacuum furnace melting	**No**	None	–N/A–	Higher elongation for Ti–Cu alloy up to 26%	**Yes**
						Tensile strength of Ti–Cu alloy after heat treatment up to 600 MPa	
[3] [49]	Ti6Al4V +**5 wt.% Cu**	Repeated melting	**No**	None	–N/A–	Yield strength: 999 MPa	**Yes**
						UTS: 1151 MPa	
						Hardness (Hv): 371	
[4] [50]	CpTi +**3, 5, 7, and 10 wt.% Cu**	SLM	**Yes**	None	–N/A–	Microhardness: Max-535 Hv	**No**
						Electrochemical corrosion tests	
[5] [51]	CpTi +**3 wt.% Cu**	Microwave sintering	**No**	None	–N/A–	Compressive strength: Max—1062 MPa	**No**
						Elastic modulus: Max-12 GPa	
						Electrochemical corrosion tests	
[6] [52]	CpTi +**5 wt.% Cu**	SLM	**Yes**	None	–N/A–	None	**None**
[7] [50]	Ti6AL4V +**5 wt.% Cu**	DED (3D systems)	**Yes**	Surface etching	–N/A–	None	**None**
[8] [53]	Ti6Al4V +**5 wt.% Cu**	DED	**Yes**	Titania nanotube on surface of Ti-Cu alloys	–N/A–	Electrochemical corrosion testing	**None**
[9] [54]	Ti6Al4V	Pre-forged Ti plates	**No**	Sputter coating Ti surface with Cu and carbon thin film	None	–N/A–	**Yes**
[10] [55]	Ti6Al4V	Commercially available Ti plates	**No**	Sol–Gel coating of copper filled-Titanium oxide	None	–N/A–	**No**

### 4.1. Modifying Ti6Al4V alloy chemistry for improved biocompatibility

Despite the superior biological performance of CpTi, the most relevant metal alloy in the biomedical implant industry remains Ti6Al4V due to its excellent mechanical performance and corrosion resistance since its adoption from the aerospace industry [56]. Currently, most implants use Ti6Al4V to provide strength and longevity with either a porous coating of a more biocompatible metal or calcium phosphates to enhance biocompatible surface characteristics [57]. Ti6Al4V exhibits *α* + *β* dual-phase microstructure, with Al as the *α*-phase stabilizer and V as the *β*-phase stabilizer. Ti6Al4V was initially designed and invented for high-temperature oxidation-resistant aircraft structural applications. The design incorporated 6 wt.% Al and 4 wt.% V [58], to achieve the desired performance, while Al and V are not essential elements needed for load-bearing implants. The question is whether we can design an alloy composition between CpTi and Ti6Al4V with biocompatibility comparable to that of CpTi with mechanical strength equivalent to Ti6Al4V. Current research in metallic implants has advanced to modifying bulk materials using ternary Ti alloys with varying stoichiometric ratios [59–62]. However, ternary alloys have drawbacks concerning materials processing difficulties and poor fatigue performance. To circumvent such roadblocks, this study aims to eliminate a dual-material bulk and surface-coating type approach and provide a superior novel binary Ti alloy, Ti3Al2V, explicitly designed for the biomedical industry in load-bearing implant applications. Our composition aims to establish a median between CpTi and Ti6Al4V via additive fabrication—CpTi’s biocompatibility and Ti6Al4V’s mechanical performance. To achieve this, Ti6Al4V and CpTi raw powders were combined in a 1:1 ratio to create Ti3Al2V. The approach assured regulatory compliance and aligned with commercial implant manufacturing requirements. By prioritizing mixing over pre-alloying, we developed an intermediate alloy between CpTi and Ti6Al4V, simplifying the alloy design process for Ti3Al2V. The fundamental component of our alloy design method, which focuses on the blending of CpTi and Ti6Al4V for specific benefits, would be missed if we used a pre-alloyed composition like Ti3Al2.5 V, even if it is commercially accessible and similar to Ti3Al2V.

Metallic implants at load-bearing sites demand high yield strength instead of ultimate tensile strength simply because biomedical implants are designed to resist complete failure. In addition, the *in vivo* applied load on the implant must stay under the yielding point in the elastic stress*–*strain region for the material to avoid permanent plastic deformation, which might create complications and lead to implant fracture. The high compressive yield strength of additively manufactured dense Ti6Al4V results from the constituting Al and V solute atoms. We observed no change in elastic modulus on reducing the solute atoms by half, but the compressive yield strength for dense Ti3Al2V was reduced by 18% without compromising the fatigue resistance. However, compared to SLM-processed CpTi (compressive yield strength = 560 MPa) [63], dense Ti3Al2V showed 72% higher yield strength. Low-modulus binary Ti alloys, such as Ti–Nb (niobium), have been evaluated for phase stability and transformation based on temperature dependence and *β* stabilizing solute addition [64]. At high temperatures, Ti alloys exhibit a transition to highly anisotropic *α*″ microstructure, which gradually transforms back to *β* either upon gradual cooling to room temperature or increase in addition of *β* stabilizing solute atoms such as Nb. The observation of acicular *α*′ martensitic microstructure in Ti3Al2V follows such findings because our alloy has compositionally reduced amounts of V, a *β* stabilizer in Ti6Al4V, and the SLM processing of this alloy involves rapid melt pool cooling as opposed to a gradual decrease to room temperature. The *α*′ lamellae observed for Ti3Al2V are primarily due to the influence of checkerboard SLM processing, where the melt pool thermal gradient varies for each layer, leading to typical basketweave microstructures for *α* + *β* Ti alloys [64]. With the addition of Ta in Ti3Al2V, we observe an increase in the modulus value. Typically, it is expected for the modulus to decrease with Ta addition to Ti since Ta is a *β*-phase stabilizer and results in the formation of high-strength-low-modulus *β*-Ti phase [15]. In this study, the modulus value increased since Ta did not completely melt as is visible from the microstructure. Higher cooling rates in SLM printing (10^4^–10^6^ K·s^−1^) [65] prevented the formation of *β*-Ti grains and no intermetallic phases. The composition showed a modulus similar to a metal-matrix composite, with a higher value since pure Ta has an elastic modulus of ∼185 GPa [66]. Porosity is essential in biomedical implants; bulk porosities reduce stress shielding, whereas surface porosities enhance early-stage tissue integration *in vivo* [67]. This study’s porous Ti3Al2V with 42.9% porosity showed 154% higher compressive yield strength over SLM-processed CpTi structures with ∼40% porosity (∼150 MPa) [68]. Considering an implant is designed not to fracture, its modulus of elasticity is the strongest determining factor for long-term physiological fixation and seamless healing for a surgically placed implant. Our data shows a modulus for porous Ti3Al2V (32.5 GPa) to be closer to natural bone (∼5–30 GPa) than commercially used alloys, which is desired and of the highest relevance. Our data shows that introducing just 20% porosity in Ti3Al2V-P20 lowers the modulus by ∼17 GPa from Ti6Al4V-P20 with 15% porosity. Thus, from the modulus and strength perspective, Ti3Al2V is significantly better than CpTi without considerable degradation in strength from Ti6Al4V. Although processing parameters do not significantly vary between Ti6Al4V and Ti3Al2V during AM operation, the chemical constitution of a reduced Al, V solute can make a significant difference in properties relevant to biomedical applications. Adding Cu increased the compressive strength of Ti3Al2V due to precipitation hardening, but Ta addition did not make much difference due to the complete solid solubility of Ta in Ti. Most importantly, lowering Al and V in Ti from Ti6Al4V to Ti3Al2V did not degrade the fatigue resistance of these alloys.

We aimed to stay in the Ti, Al, and V domains and try to develop an alloy with improved mechanical properties. CpTi and Ti6Al4V are commercially accepted worldwide-used materials. Alloying elements as seen in *β*-Ti alloys were not used, making Ti3Al2V abide by regulatory merits for commercial use. Therefore, although Ti–24Nb–4Zr–8Sn, Ti–42Nb, and Ti–15Ta–5.5Zr displayed lower modulus and similar yield strength to Ti3Al2V, the aforementioned reasons give Ti3Al2V an added advantage over *β*-Ti alloys. Furthermore, the application of Ti3Al2V alloys can be expanded beyond primary implant material to coatings depending on the customization need of the end user. Additive manufacturing is equipped to print a porous metallic layer along with the implant in a single process and of the same composition to introduce a more uniform phase in the melt pool between adjacent layers. We observed a 17%–20% (relatively insignificant) decrease in shear strength with reduced Al and V in Ti3Al2V at the porous-dense interface, given that Ti3Al2V had 4%–7% higher porosity than Ti6Al4V. While metal-on-metal coatings are well adhered due to a single manufacturing process, calcium phosphate coating failures via cracking or delamination due to poor adhesion can sometimes lead to unsuccessful implantation [69, 70]. This makes porous metallic coatings a safer option with ease of manufacturing in a single step than calcium phosphate coatings, which generally require a second processing step. Ti3Al2V alloy meets clinical demand without compromising mechanical properties and successfully bridges the manufacturing and biomedical industries toward potential bench-to-bedside products.

### 4.2. Incorporation of Cu in Ti3Al2V as an antimicrobial agent

Copper (Cu) is the first metallic antibacterial agent authorized by the U.S. Environmental Protection Agency due to its antibacterial efficacy. Copper’s antibacterial properties are influenced mainly by its concentration, oxidation state (Cu^0^, Cu^1+^, or Cu^2+^), and form (ion or nanoparticle). Moreover, the application method (dry or wet), ambient temperature, and the contact distance between microbes and surfaces containing Cu influence its antibacterial activity [71]. Regardless of the form, contact killing – the most common phenomenon of contact with bacteria on Cu-containing surfaces – is one of the critical mechanisms of Cu’s antibacterial impact. Other mechanisms include cell membrane disruption, reactive oxygen species production, and contact toxicity. When bacteria comes into close contact with surfaces that contain Cu, the bacteria is quickly eliminated. Effective exposure time is shorter in a dry environment and longer in a damp setting [72]. Current research indicates that Cu ions from Cu-containing surfaces bind to negatively charged peptidoglycans in the outer membrane of bacterial cells, leading to the leakage of the cell’s contents called cytoplasmic outflow process. This is followed by inhibiting the intracellular respiratory chain activity, which causes oxidative stress damage and apoptosis [49, 73]. The mechanisms underlying contact killing are still poorly understood. Since we focused on comprehensively evaluating the first generation of Ti3Al2V alloys for biomedical application, studying potential bacterial resistance upon adding Cu was imperative from a clinical perspective. Our results reflect similar characteristics for bacterial contact-killing. Percent bacterial viability for *S. aureus* on Ti3Al2V–3Cu surfaces displayed significant contact-killing as early as 24 h up to 48 h compared to CpTi and Ti6Al4V control compositions. A similar trend was observed for gram-negative *P. aeruginosa* over 36 h of exposure to Cu-containing Ti3Al2V alloys. Moreover, SEM micrographs of surfaces for Ti3Al2V–2Cu and Ti3Al2V–3Cu show septum formation for *S. aureus* and cytoplasmic outflow for both bacterial strains, which is morphological evidence of the antibacterial efficiency of the Ti3Al2V–Cu alloys [74]. The reduction of planktonic bacteria adhered on the Ti3Al2V surface can correlate to the antibacterial effect of TiO_2_ photocatalytic property. Considering that the Ti matrix increases when alloying CpTi and Ti6Al4V while reducing the Al and V ratio, it can significantly increase the self-passivation of Ti via TiO_2_ layering [75]. Ultimately, all the above processes in synergistic combination result in cell lysis through cytoplasmic outflow from the highly permeable cell membrane of the bacterial cell and fortify the biomaterial surface for osseous tissue integration.

### 4.3. Synergistic enhancement in the biocompatibility of Ti–Cu alloys

Over the past few decades, many ternary *β*-Ti alloys have been developed to replace Ti6Al4V in biomedical applications for improved mechanical properties and biocompatibility. More elaborately, such alloy development was initiated to attain lower modulus and higher biocompatibility than generic Ti via biocompatible alloying elements. However, evidence shows that *β*-Ti alloys do not offer any significant compositional advantage in early-stage biological performance compared to CpTi and Ti6Al4V. Although data available in the literature provides several ternary alloys that can have potential applications as biomaterials [76–79], the overall evaluation does not provide a strong enough argument to legitimize them as a replacement for commercially available, CpTi or Ti6Al4V, despite their drawbacks. Considering Ta and Cu are *β*-Ti phase stabilizers, we should expect the formation of high-strength, low-modulus *β*-Ti with the addition of Ta and Cu in Ti3Al2V [80]. Previous studies have shown results where LENS-manufactured Ti–Ta compositions show lower elastic modulus than pure Ti due to the formation of the *β*-Ti phase [15]. However, the compression test results indicate an increase in the elastic modulus compared to Ti3Al2V. For dense Ti3Al2V–10Ta–3Cu composition with a residual vol. porosity of 3.7%, we observe the elastic modulus to be 127.6 GPa as opposed to that for dense Ti3Al2V (114.1 GPa). Ti–Ta–Cu, with a volume porosity of 15.2% showed an elastic modulus of 113.3 GPa, equivalent to that for dense Ti3Al2V. Comparing the compressive yield strengths, we also observe increased strength values for the Ti3Al2V–10Ta–3Cu compositions from Ti3Al2V. Dense Ti3Al2V–10Ta–3Cu displayed a compressive yield strength of 1271 MPa, whereas it was 965 GPa for Ti3Al2V. The compressive strength observed for Ti3Al2V–10Ta–3Cu with 15.2% porosity was 1010 MPa, even higher than that for dense Ti3Al2V. It is hypothesized that higher cooling rates in the SLM process (10^4^–10^6^ K·s^−1^) [65] compared to that of the DED process (10^3^–10^5^ K·s^−1^) [81] resulted in a lack of formation of the *β*-Ti phase with no intermetallic formation in SLM processed compositions than DED processed Ti–Ta in Mitra *et al* [15]. The enhancement in strength is primarily due to Cu addition to Ti3Al2V due to precipitation hardening.

### 4.4. Tribological analysis

Bioactivity in metallic biomaterials occurs through the natural formation of a passivation layer on the surface or by the intentional surface coating during manufacturing; this passivation layer or coating may wear out over the implant’s life or operation for load-bearing articulating surfaces, revealing the bulk underlying parent material. If an improper material is used, this may promote adverse physiological reactions or interfere with the cell’s resting potential. The cell’s resting potential, as it is most commonly referred to, is known as the electrochemical potential difference along the cell membrane governed by ion concentrations, namely by the balance of Na^+^ and K^+^ [82]. Literature has reported the physiological resting potential to be ∼−73 mV [83]; this value is most commonly derived using the Nernst equation [84]. Measuring the OCP during tribological testing allows for an improved understanding of the material-tissue interaction, in terms of the electrochemical match or mismatch. Considering the body’s resting potential, a more cathodic (positive) material, nearing the physiological resting potential, would have the least tendency to behave as an anode and thus reduce the tendency for corrosion to occur. Typical scenarios are observed in the marine industry; cathodic protection by use of a sacrificial metal, such as zinc is used to protect bridges, barges, and ships from corrosion. The sacrificial metal, displaying a more anodic standard reduction potential, when placed on the material to be protected preferentially corrodes. For this reason, a more cathodic material is sought as an implant material. In the present study, it was observed that CpTi under idle conditions was the noblest composition and above the −73 mV physiological resting potential, figure 3. Ti3Al2V Cu-containing compositions exhibited OCP values under idle conditions at ∼ −73 and −76 mV, for Ti3Al2V–10Ta–3Cu and Ti3Al2V–3Cu, respectively. Interpreting these OCP (*E*_Idle_) values concludes that the two aforementioned compositions would have the least tendency to influence an imbalance of the physiological ion concentration across the cell membrane. During tribological testing, Ti3Al2V–10Ta–3Cu and Ti3Al2V–3Cu shifted to a more cathodic potential when compared to the remaining four compositions. A positive shift in OCP would be ideal for a material in contact with the physiological environment as the tendency for negative physiological effects may be reduced. When the tribological testing was concluded, it was observed that Ti3Al2V–3Cu, Ti3Al2V–10Ta, and Ti3Al2V–10Ta–3Cu re-passivated to more cathodic OCP values when compared to the remaining three compositions. Ti3Al2V–3Cu re-passivated quicker than Ti3Al2V–10Ta and observed in Ti3Al2V–10Ta–3Cu was a median of re-passivation when considering the first two mentioned compositions.

Materials for load-bearing articulating sites should also exhibit fitting wear resistance as this goes hand-in-hand with reducing wear debris, which would otherwise be free to react and promote adverse physiological effects [85, 86]. Evaluation of the wear regime for two materials in tribological contact can become convoluted, even simply by altering the two materials or the media of choice [87, 88]; two materials can exhibit desired synergistic wear modes in one media and then deviate to catastrophic failure under tribological conditions in a different media. Titanium inherently displays a low resistance to plastic shear as it is a passive metal, displaying both cohesive and adhesive dynamic failure [89]; this is observed in the wear scar imaging of CpTi. Analyzing the wear scar it was revealed that plastic deformation, gouging, material removal, and subsequent deposition of the removed material occurred. Alternatively, observed across Ti3Al2V–3Cu, Ti3Al2V–10Ta, and Ti3Al2V–10Ta–3Cu was the reduction in wear scar on the counter wear ball. A reduction of material transfer was observed; therefore, a reduction in cohesive failure and adhesive material transfer to the counterwear ball occurred due to Cu and Ta addition.

### 4.5. Improving biocompatibility of Ti3Al2V with Cu and Ta addition

The entire load-bearing biomedical research focuses on developing biocompatible metal alloys to display improved tissue integration [90, 91]. Knowing early-stage osseointegration significantly affects an implant’s eventual *in vivo* fixation and the patient-healing period, especially for those with suboptimal bone quality, poor osseointegration may lead to implant loosening and, eventually, revision surgery [92]. Our study employs a bilateral approach toward evaluating the biocompatibility of Ti3Al2V–10Ta–3Cu functional alloy design. First, the *in vivo* biological response of Ti3Al2V alloy was evaluated and compared with its counterparts—CpTi and Ti6Al4V. Histological analysis of implant-bone sections revealed no evidence of inflammatory response or infection for these porous implants for all compositions. Observations of uniform and improved tissue integration, osteoid formation inside the interconnected pore channels, and trabecular bone formation very well adjacent to the outer surface of the implant were concluded for Ti3Al2V. Although CpTi has few focal trabecular bone growth regions, the continuous lining of fibrocartilage, along with the implant interface and complete fibrocartilage infiltration into the implant’s pores, makes CpTi comparatively inferior to Ti3Al2V. The H&E section for CpTi displayed tissue integration in isolated areas, which mainly consisted of osteoblastic tissue proximal to the implant surface with distal osteoid formation. Alternatively, Ti3Al2V displayed very well-integrated trabecular bone (dark purple) along the implant surface with evidence of connective tissue embedded in the osteoblastic matrix, indicating early phases of vessel/capillary formation. Ti6Al4V implant-bone section exhibited no evidence of tissue integration or bone mineralization fronts. The presence of definite gaps along the edge of the implant aids in distinguishing between the *in vivo* biological integration between Ti6Al4V and the other two compositions. The trabecular bone observed at the BIC of the Ti6Al4V implants reflects residual bone fragments adjacent to the implant’s surface during surgical placement, evident from their continuation from the outer cortical bone as well as fibrous tissue (dark blue lining) extending from the lateral edge of the implant, figures 4, 5 and supplemental figure S1. It should be noted that fibrocartilage lining, or formation, is not an adverse physiological reaction toward the implant material. Although fibrocartilage can transmit loads, in this study, a uniform fibrocartilage presence across all compositions only reflects that the trabecular bone formed at 6 weeks post-implantation is mostly woven bone developing from the fibrocartilage.

Once we had established the superior biocompatibility of Ti3Al2V alloys, we determined the biological host tissue response on Ti3Al2V–3Cu and Ti3Al2V–10Ta–3Cu alloys compared to Ti3Al2V–10Ta alloy, which was based on our previous reports is considered as a positive control. Ti3Al2V–3Cu displayed osteoid lining in very close proximity to the implant surface, along with areas of interwoven lamellar bone into the implant and the fibrocartilage lining that runs through the central porous channel of the implant. These results establish Ti3Al2V–3Cu to be equally compatible with CpTi. No neoplasia, inflammation, or bone densification was observed as a product of Cu alloying with Ti. Upon incorporation of 10 wt.% Ta in Ti3Al2V–3Cu alloys, Ti3Al2V–10Ta–3Cu alloys exhibited a significant shift of fibrocartilaginous matrix to the interwoven lamellar bone in the porous channels of the implant and very well-formed osteoid lining with osteoblastic cells at the intercondylar fossa that have not yet been initiated into bone remodeling and osteoid formation. Such observations restore this composition toward our positive control group Ti3Al2V–10Ta which exhibits remarkable bone formation and tissue mineralization even into the porous channels of the implant. Overall, we have not only successfully developed a novel binary alloy of CpTi and Ti6Al4V but incorporated bacterial inhibition with the addition of 3 wt.% Cu and further enhanced biocompatibility by adding 10 wt.% Ta, which comprehensively shows early-stage *in vivo* performance better than its counterparts at only 6 weeks post-implantation.

## 5. Conclusions

Septic loosening of orthopedic implants has been a rising adverse clinical outcome that significantly affects all the 5 Ps’ of implant intervention for orthopedic surgeries—patients, physicians, payers, policies, and product manufacturers. Mitigating microbial infection-assisted septic loosening of implants is particularly important because it affects outcomes across the board, whether THAs, TKAs, spinal devices or fracture fixation devices. Despite having highly heterogeneous modes of device implantation and failure, any adverse outcome is measured using the same yardstick—increasing healthcare costs—since most health insurance does not cover preventative outcomes and decreases the product’s value. Seemingly, overcoming these adversities is a multi-factorial problem that requires a multi-disciplinary approach to solve the problem. Overall, a promising solution would be a functional material that is universal in applicability as well as mitigates multiple drawbacks responsible for poor biocompatibility. Our research on novel Ti3Al2V alloy development leading to a more promising Ti3Al2V–10Ta–3Cu alloy has demonstrated characteristics of multi-functionality. We have improved biocompatibility for Ti3Al2V compared to its counterparts, CpTi and Ti6Al4V, by minimizing the V and Al components from Ti6Al4V—which add no significant biological properties to these alloys. Minimizing V and Al from the material’s chemistry did not compromise the mechanical properties of the novel Ti3Al2V alloy. Furthermore, adding only 3 wt.% Cu to Ti3Al2V aided in imparting microbial resistance functionality to Ti3Al2V alloys against the two most high-risk bacterial strains *S. aureus* and *P. aeruginosa* with antibacterial efficacy 80% higher than CpTi and Ti6Al4V. As discussed, our research comprehensively tackles a multi-factorial problem. Considering bacterial colonization deteriorates implant biocompatibility, adding Cu could alleviate that condition; however, Cu could also alter bone response when implanted *in vivo*. Therefore, we explored the extent of alterations and established an alternative to mitigate any compromise in biocompatibility. Our best-performing Ti3Al2V–10Ta–3Cu alloys are binary alloys that offer microbial resistance, enhanced biomechanical performance, and enhanced biocompatibility compared to CpTi, Ti6Al4V, Ti3Al2V–3Cu alloys alone. In summary, thousands of patients have reported implant failures due to external causes such as microbial infection, which a Ti6Al4V implant is not tailored to overcome. We feel a material like Ti3Al2V–10Ta–3Cu could only improve patients’ postoperative quality of life and reduce revisional surgeries that arise from multiple scenarios, including bacterial infection.

## Data Availability

All raw data for this study has been presented in this manuscript.
